# Supplementary material for: Structural insights into the potency and selectivity of covalent pan-FGFR inhibitors
Source: Commun Chem. 2022 Jan 11;5:5. doi: 10.1038/s42004-021-00623-x (PMC9814232; doi:10.1038/s42004-021-00623-x)
Supplement: Supplementary file 1 — Supplementary Information [file 42004_2021_623_MOESM1_ESM.pdf]

Supplementary information

Structural insights into the potency and selectivity of covalent pan-FGFR inhibitors

Supplementary Table 1: Data collection and refinement statistics

	SRC/FIIN-2	SRC/TAS-120	FGFR4/PRN1371
**Data collection**			
Space group	P 1	P 1	P 1 21 1
Cell dimensions			
a, *b*, *c* (Å)	41.8 63.0 74.1	42.0 63.2 73.1	42.4 61.1 61.0
α, β, γ (°)	79.0 90.0 90.0	100.6 90.6 89.8	90.0 97.7 90.0
Resolution (Å)	26.7-2.1(2.2-2.1)	28.9-2.7 (2.8-2.7)	43.0-2.3 (2.4-2.3)
R_{sym} or R_{merge}	0.091 (0.60)	0.12 (0.46)	0.092 (0.24)
$I / \sigma I$	11.5 (0.97)	9.3 (2.4)	7.9 (2.9)
Completeness (%)	95.5 (86.9)	96.1 (73.4)	92.2 (66.1)
Redundancy	3.0 (2.3)	3.3 (2.4)	6.4 (6.7)
**Refinement**			
Resolution (Å)	26.7-2.1	28.9-2.7	43.0-2.3
No. reflections	35291	19828	13003
R_{work} / R_{free}	0.19/0.23	0.18/0.23	0.19/0.24
No. atoms			
Protein	4082	3974	2348
Ligand/ion	104	62	53
Water	284	56	139
B-factors			
Protein	43.4	53.0	35.4
Ligand/ion	59.1	73.7	48.8
Water	42.0	45.8	36.4
R.m.s. deviations			
Bond lengths (Å)	0.003	0.005	0.003
Bond angles (°)	0.6	0.9	0.76
PDB code	7D57	7D5O	7F3M

Supplementary Table 2: List of all primers

FGFR1 clone to pET28a vector
FGFR1 F: GTTCTGTTTCAAGGCCCGGCAGGGGTCTCTGAGTAT<br>FGFR1 R: GTGGTGGTGCTCGAGTTACTCCTGGTTGGAGGTCAAG
FGFR2 clone to pET28a vector
FGFR2 F: GTTCTGTTTCAAGGCCCGGACACCCCCATGCTGGCAG<br>FGFR2 R: GTGGTGGTGCTCGAGTTATTCCTCATTGGTTGTGAG
FGFR3 clone to pET28a vector
FGFR3 F: GTTCTGTTTCAAGGCCCGACGCTGGCCAATGTCTC<br>FGFR3 R: GTGGTGGTGCTCGAGTTAGTCGGTGGACGTCACGGT
FGFR4 clone to pET28a vector
FGFR4 F: GTTCTGTTTCAAGGCCCGTTGCTCGCCGGCCTCGTG<br>FGFR4 R: GTGGTGGTGCTCGAGTTACTCCTCAGAGACGGCCAG
SRC clone to pET28a vector
SRC F: GTTCTGTTTCAAGGCCCGCAGACCCAGGGACTC<br>SRC R: GTGGTGGTGCTCGAGTTATAGGTTCTCTCCAGGCTG
HCK clone to pET28a vector
HCK F:GTTCTGTTTCAAGGCCCGCAGAAGCCTTGGGAGAAAGA<br>HCK R:GTGGTGGTGCTCGAGTTATCATGGCTGCTGTTGGTACT
Mutations
SRC(C277A) F: GAAGCTGGGGCAGGGCTGCTTTGGAGAGGTCTGGATG<br>SRC(C277A) R: CATCCAGACCTCTCCAAAGCAGCCCTGCCCCAGCTTC
FGFR1(V561M) F: GTCCCTTGTATGTCATCATGGAGTATGCCTCCAAGG<br>FGFR1(V561M) R: CCTTGGAGGCATACTCCATGATGACATACAAGGGAC
FGFR2(V564F) F: CCTCTCTATGTCATATTCGAGTATGCCTCTAAAG<br>FGFR2(V564F) R: CTTTAGAGGCATACTCGAATATGACATAGAGAGG
FGFR3(V555M) F: C CCCCTGTACGTGCTGGTGGAGTACGCGGCCAAG<br>FGFR3(V555M) R: CTTGGCCGCGTACTCCACCAGCACGTACAGGGG
FGFR4(V550L) F: GGGCCCCTGTACGTGATCCTGGAGTGCGCCGCCAAGG<br>FGFR4(V550L) R: CCTTGGCGGCGCACTCCAGGATCACGTACAGGGGCCC
FGFR4(C477A) F: CCCCTAGGCGAGGGCGCCTTTGGCCAGGTAGTAC<br>FGFR4(C477A) R: GTACTACCTGGCCAAAGGCGCCCTCGCCTAGGGG

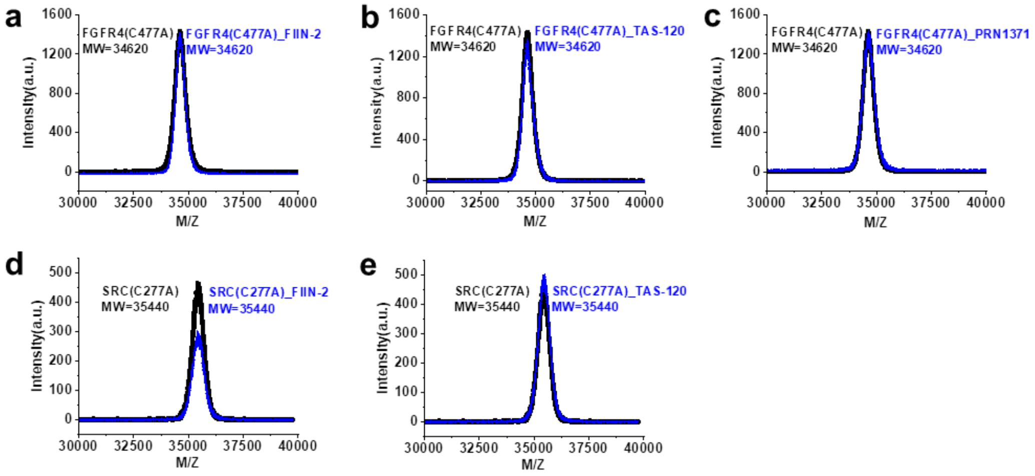

Supplementary Fig. 1: Binding modes of FIIN-2, TAS-120 and PRN1371 to FGFR4(C477A) and SRC(C277A).

a-c MALDI-TOF MS of apo FGFR4(C477A) (black) and FGFR4(C477A)/inhibitor mixture (blue). **d-e** MALDI-TOF MS of apo SRC(C277A) (black) and SRC(C277A)/inhibitor mixture (blue).

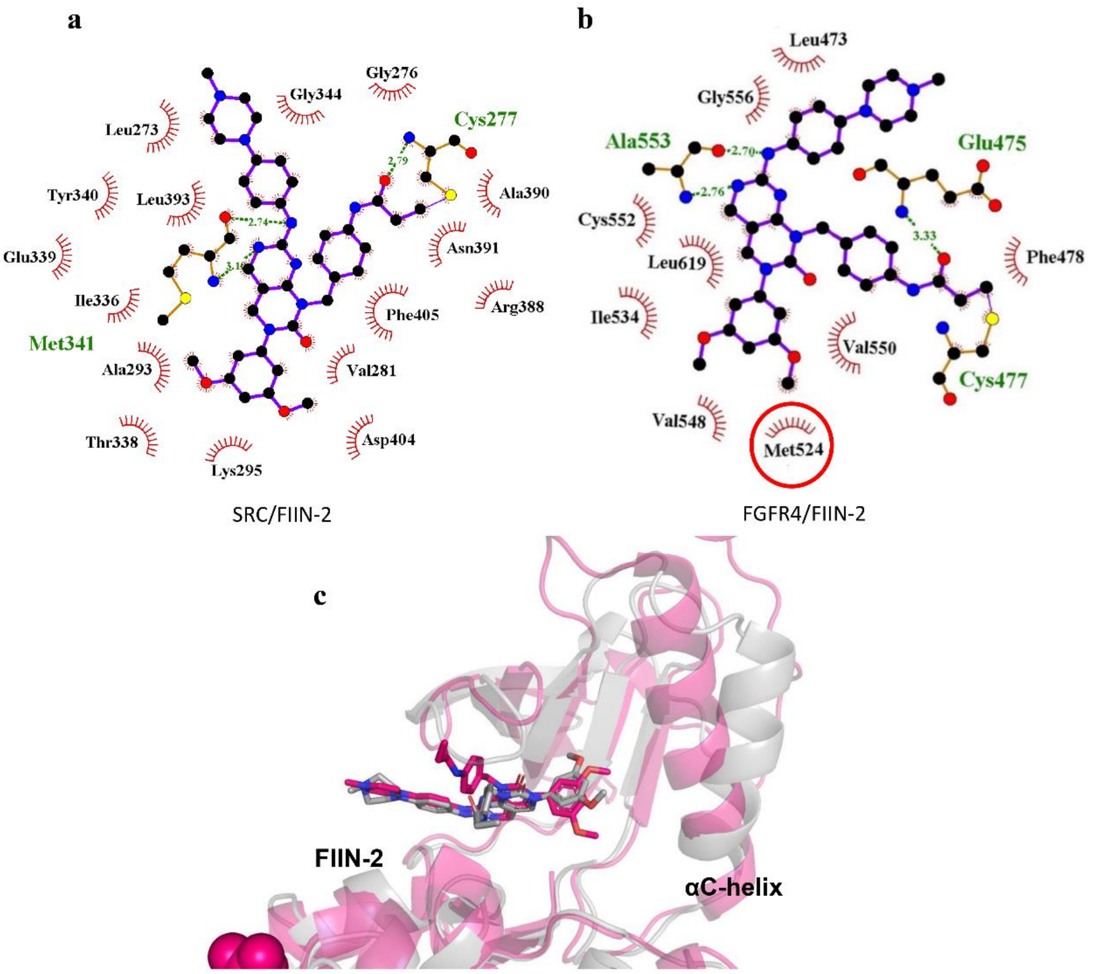

Supplementary Fig. 2: Binding modes of FIIN2 with SRC and FGFR4.

Ligand interaction diagram (LID) demonstrated the detailed interactions of SRC/FIIN-2 (**a**), FGFR4/FIIN-2 (PDB: 4QQC) (**b**). Hydrogen bonds are designated with a green dashed line, and van der Waals interactions are shown by a red half "sun" arrangement. The red circle circles are the interaction with αC-helix. Graphics were drawn by LigPlot. **c** Superposition of SRC/FIIN-2 structure with FGFR4/FIIN-2(PDB:4QQC). FGFR4/FIIN-2 is colored pink; SRC/FIIN-2 is colored grey.

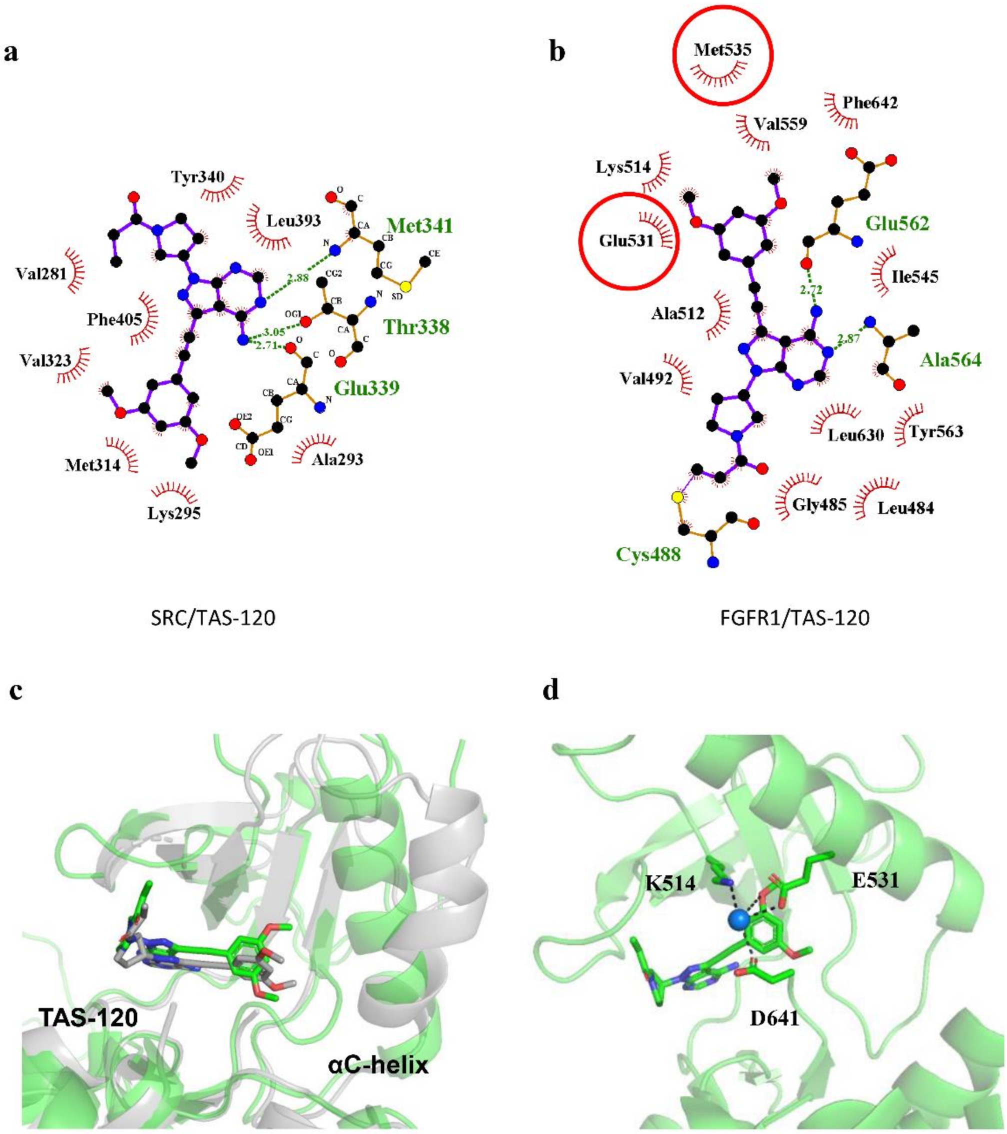

Supplementary Fig. 3: Binding modes of TAS-120 with SRC and FGFR1.

Ligand interaction diagram (LID) demonstrated the detailed interactions of SRC/ TAS-120 (**a**), FGFR4/TAS-120 (PDB: 6MZW) (**b**). **c** Superposition of the SRC/TAS-120 structure with FGFR1/TAS-120(PDB:6MZW). FGFR1/TAS-120 is colored green; SRC/TAS-120 is colored grey; **d** Water-mediated hydrogen bonds in the FGFR1/TAS-120 complex. Water is shown as blue sphere.

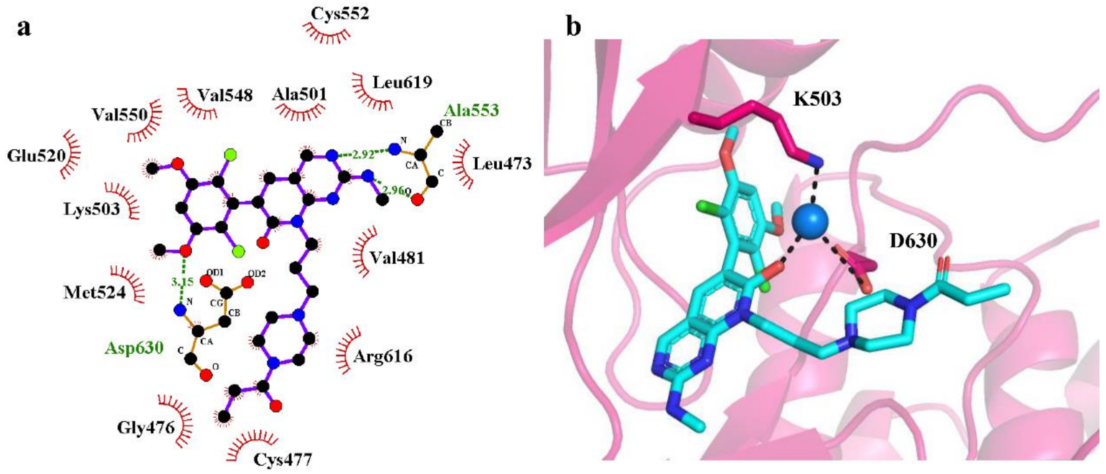

Supplementary Fig. 4: Binding modes of PRN1371 with FGFR4.

a Ligand interaction diagram (LID) demonstrated the interaction of PRN1371/FGFR4 complex. Hydrogen bonds are designated with a green dashed line, and van der Waals interactions are shown by a red half "sun" arrangement. Graphics were drawn by LigPlot. **b** The water-mediated hydrogen bonds in the FGFR4/PRN1371 complex. The water is shown as blue sphere.

a

b

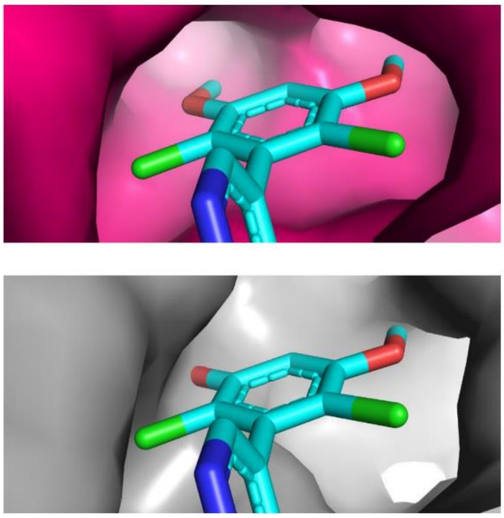

Supplementary Fig. 5: Comparison of the binding pocket of FGFR4 and SRC.

a Surface representation of FGFR4 shows the binding pocket for PRN1371. **b** Surface representation of SRC shows the binding pocket. The modelled SRC/PRN1371 structure was acquired by a superposition of SRC/FIIN-2 structure with the FGFR4/PRN1371 structure. FGFR4 is colored pink; SRC is colored grey; PRN1371 is shown as cyan stick.

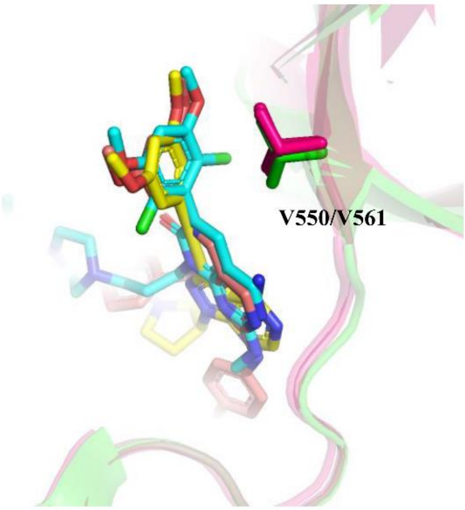

Supplementary Fig. 6: Superposition of the structures of FGFRs in complex with inhibitors.

Superposition of FGFR4/FIIN-2 structure (PDB:4QQC) with FGFR1/TAS-120 (PDB:6MZW) and FGFR4/PRN1371 structure. FGFR1 is colored green; FGFR4 is colored pink; FIIN-2 is shown as salmon stick; TAS-120 is shown as yellow stick; PRN1371 is shown as cyan stick.

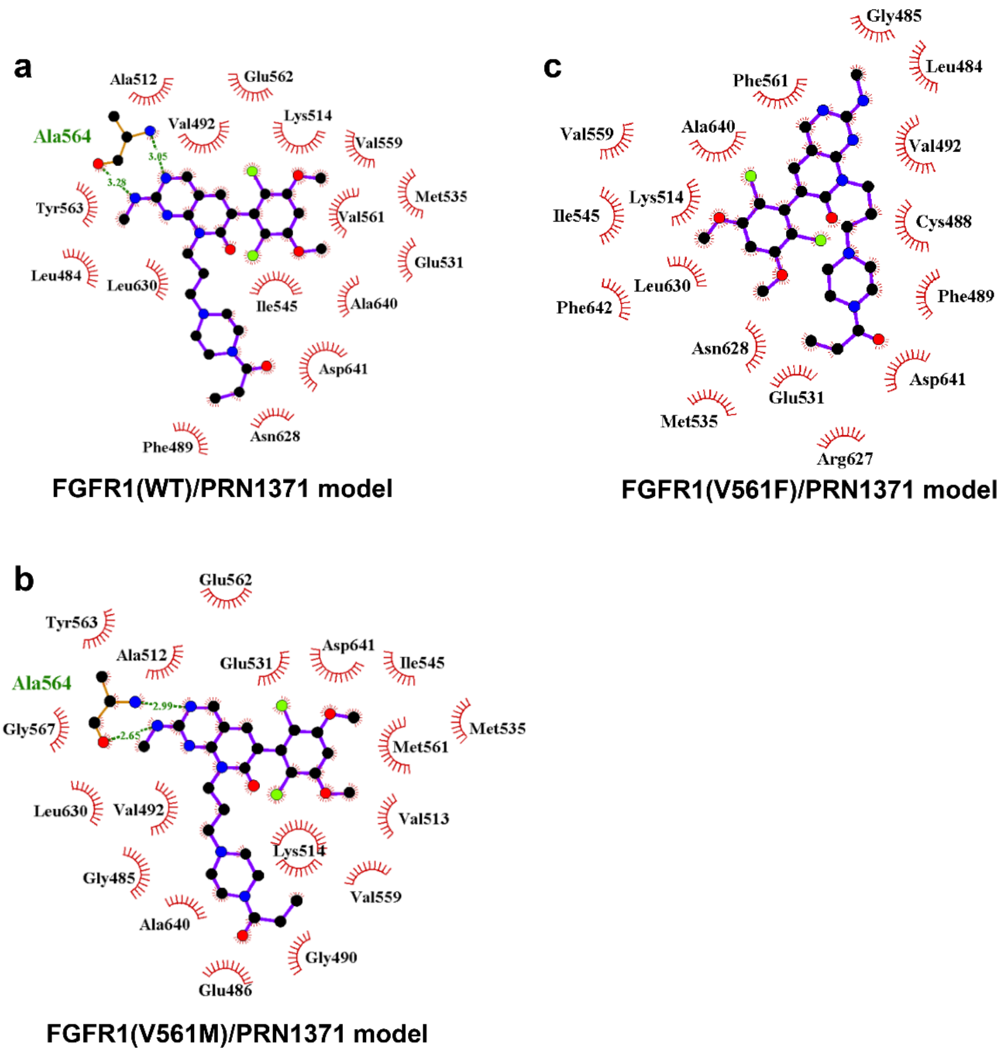

Supplementary Fig. 7: Ligand interaction diagram (LID) demonstrated the interaction of PRN1371 with FGFR1 and the gatekeeper mutant.

a Docking of PRN1371/FGFR1 (WT) complex, the interaction patten is similar with PRN1371/FGFR4; **b** Docking of PRN1371/FGFR1 (V561M); **c** Docking of PRN1371/FGFR1 (V561F). Hydrogen bonds are designated with a green dashed line, and van der Waals interactions are shown by a red half "sun" arrangement. Graphics were drawn by LigPlot.

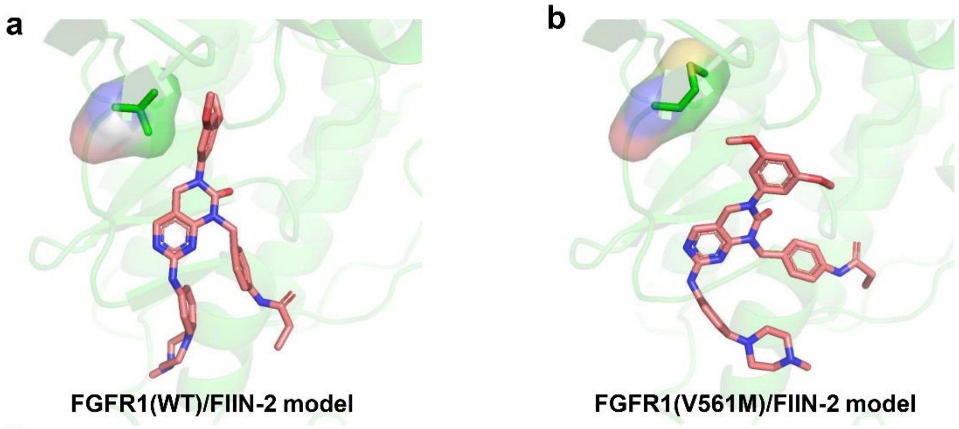

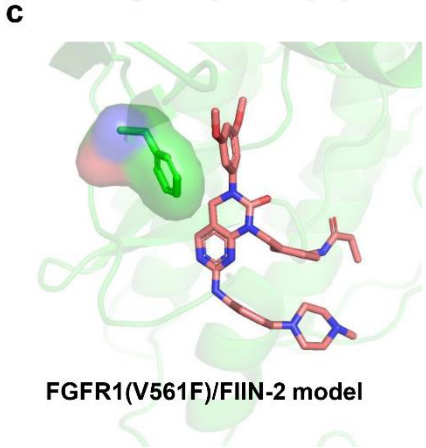

d

	WT	V561M	V561F
**Predicted Binding Energy (Kcal·mol^{-1})**	-8.1	-8.1	-7.11

Supplementary Fig. 8: Structural models of FGFR1 gatekeeper mutants with FIIN-2. Docking model of FIIN-2 to FGFR1(WT) (**a**), FGFR1(V561M) (**b**) and FGFR1(V561F) (**c**). **d** Predicted binding energies of FGFR1 gatekeeper mutants with FIIN-2. Molecular docking was performed by AutoDock Tools program.

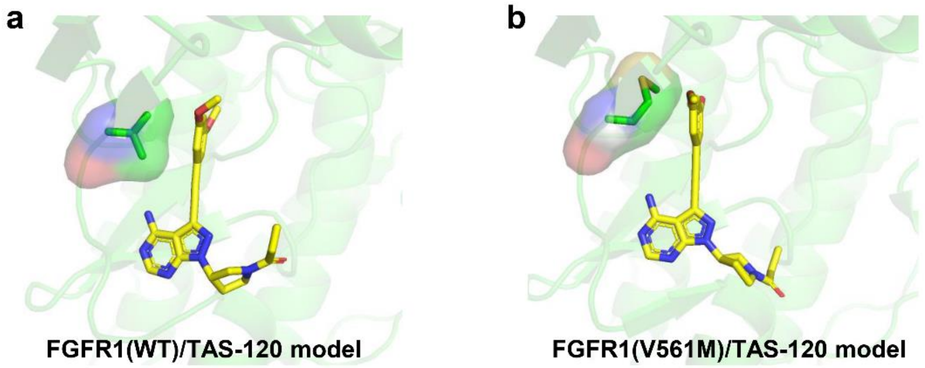

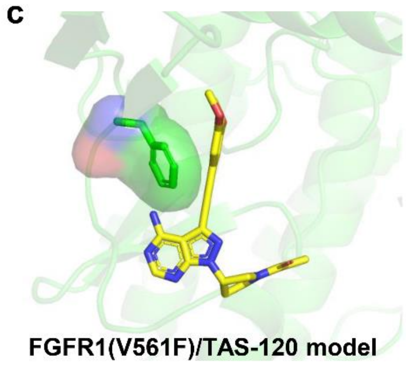

d

	WT	V561M	V561F
**Predicted Binding Energy (Kcal·mol^{-1})**	-8.6	-8.4	-7.9

Supplementary Fig. 9: Structural models of FGFR1 gatekeeper mutants with TAS-120. Docking model of TAS-120 to FGFR1(WT) (**a**), FGFR1(V561M) (**b**) and FGFR1(V561F) (**c**). **d** Predicted binding energies of FGFR1 gatekeeper mutants with TAS-120. Molecular docking was performed by AutoDock Tools program.
