# Supplementary material for: Structural insights into the potency and selectivity of covalent pan-FGFR inhibitors
Source: Commun Chem. 2022 Jan 11;5:5. doi: 10.1038/s42004-021-00623-x (PMC9814232; doi:10.1038/s42004-021-00623-x)
Supplement: Supplementary file 3 — Description of Additional Supplementary Files [file 42004_2021_623_MOESM3_ESM.pdf]

Description of Additional Supplementary Files

File Name: Supplementary Data 1

Description: Validation report of the PDB
