# Supplementary material for: Structural insights into the potency and selectivity of covalent pan-FGFR inhibitors
Source: Commun Chem. 2022 Jan 11;5:5. doi: 10.1038/s42004-021-00623-x (PMC9814232; doi:10.1038/s42004-021-00623-x)
Supplement: Supplementary file 4 — Supplementary Data 1 [file 42004_2021_623_MOESM4_ESM.zip › validation report/D_1300018767_val-report-full_P1.pdf]

WORLDWIDE PDB PROTEIN DATA BANK

Full wwPDB X-ray Structure Validation Report (i)

May 28, 2021 – 04:29 PM JST

PDB ID	:	7D57
Title	:	C-Src in complex with FIIN-2
Deposited on	:	2020-09-25
Resolution	:	2.10 Å(reported)

This is a Full wwPDB X-ray Structure Validation Report.

This report is produced by the wwPDB biocuration pipeline after annotation of the structure.

We welcome your comments at *validation@mail.wwpdb.org*
A user guide is available at
https://www.wwpdb.org/validation/2017/XrayValidationReportHelp
with specific help available everywhere you see the (i) symbol.

The following versions of software and data (see references (i)) were used in the production of this report:

MolProbity	:	4.02b-467
Mogul	:	1.8.5 (274361), CSD as541be (2020)
Xtriage (Phenix)	:	1.13
EDS	:	2.18
buster-report	:	1.1.7 (2018)
Percentile statistics	:	20191225.v01 (using entries in the PDB archive December 25th 2019)
Refmac	:	5.8.0158
CCP4	:	7.0.044 (Gargrove)
Ideal geometry (proteins)	:	Engh & Huber (2001)
Ideal geometry (DNA, RNA)	:	Parkinson et al. (1996)
Validation Pipeline (wwPDB-VP)	:	2.18

CONFIDENTIAL VALIDATION REPORT

1 Overall quality at a glance (i)

The following experimental techniques were used to determine the structure:
X-RAY DIFFRACTION

The reported resolution of this entry is 2.10 Å.

Percentile scores (ranging between 0-100) for global validation metrics of the entry are shown in the following graphic. The table shows the number of entries on which the scores are based.

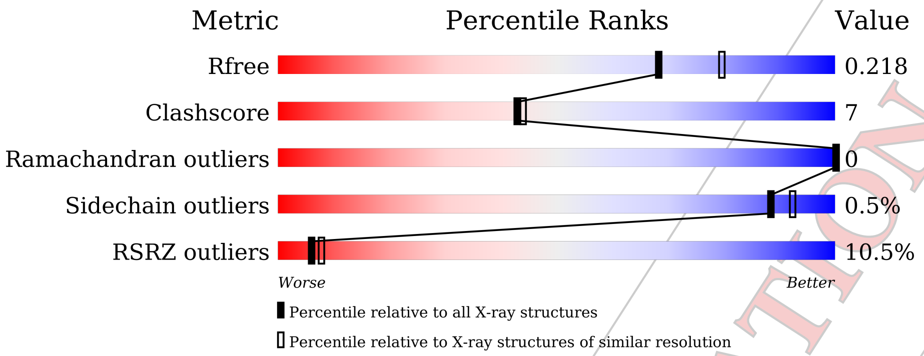

Metric	Whole archive (#Entries)	Similar resolution (#Entries, resolution range(Å))
R_{free}	130704	5197 (2.10-2.10)
Clashscore	141614	5710 (2.10-2.10)
Ramachandran outliers	138981	5647 (2.10-2.10)
Sidechain outliers	138945	5648 (2.10-2.10)
RSRZ outliers	127900	5083 (2.10-2.10)

The table below summarises the geometric issues observed across the polymeric chains and their fit to the electron density. The red, orange, yellow and green segments of the lower bar indicate the fraction of residues that contain outliers for >=3, 2, 1 and 0 types of geometric quality criteria respectively. A grey segment represents the fraction of residues that are not modelled. The numeric value for each fraction is indicated below the corresponding segment, with a dot representing fractions <=5% The upper red bar (where present) indicates the fraction of residues that have poor fit to the electron density. The numeric value is given above the bar.

Mol	Chain	Length	Quality of chain
1	A	286	9% / 84% 8% 9%
1	B	286	10% / 72% 16% 12%

CONFIDENTIAL VALIDATION REPORT

2 Entry composition (i)

There are 4 unique types of molecules in this entry. The entry contains 4477 atoms, of which 0 are hydrogens and 0 are deuteriums.

In the tables below, the ZeroOcc column contains the number of atoms modelled with zero occupancy, the AltConf column contains the number of residues with at least one atom in alternate conformation and the Trace column contains the number of residues modelled with at most 2 atoms.

- Molecule 1 is a protein called Proto-oncogene tyrosine-protein kinase Src.

Mol	Chain	Residues	Atoms					ZeroOcc	AltConf	Trace
1	A	261	Total 2058	C 1320	N 344	O 377	S 17	0	0	0
1	B	253	Total 2012	C 1298	N 332	O 367	S 15	0	0	0

There are 6 discrepancies between the modelled and reference sequences:

Chain	Residue	Modelled	Actual	Comment	Reference
A	248	GLY	-	expression tag	UNP P00523
A	249	HIS	-	expression tag	UNP P00523
A	250	MET	-	expression tag	UNP P00523
B	248	GLY	-	expression tag	UNP P00523
B	249	HIS	-	expression tag	UNP P00523
B	250	MET	-	expression tag	UNP P00523

- Molecule 2 is N-(4-{[3-(3,5-dimethoxyphenyl)-7-{[4-(4-methylpiperazin-1-yl)phenyl]amino}-2-oxo-3,4-dihydropyrimido[4,5-d]pyrimidin-1(2H)-yl]methyl}phenyl)propanamide (three-letter code: 37O) (formula: $C_{35}H_{40}N_8O_4$) (labeled as "Ligand of Interest" by depositor).

CONFIDENTIAL VALIDATION REPORT

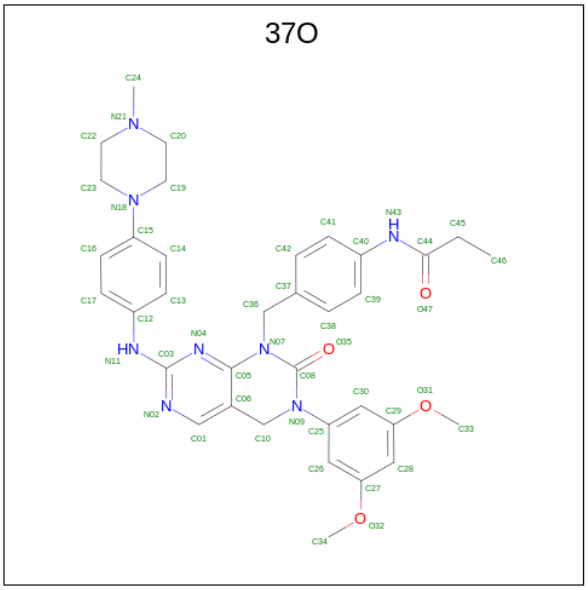

Mol	Chain	Residues	Atoms	ZeroOcc	AltConf
2	A	1	Total C N O<br>47 35 8 4	0	0
2	B	1	Total C N O<br>47 35 8 4	0	0

- Molecule 3 is SULFATE ION (three-letter code: SO4) (formula: O_4S).

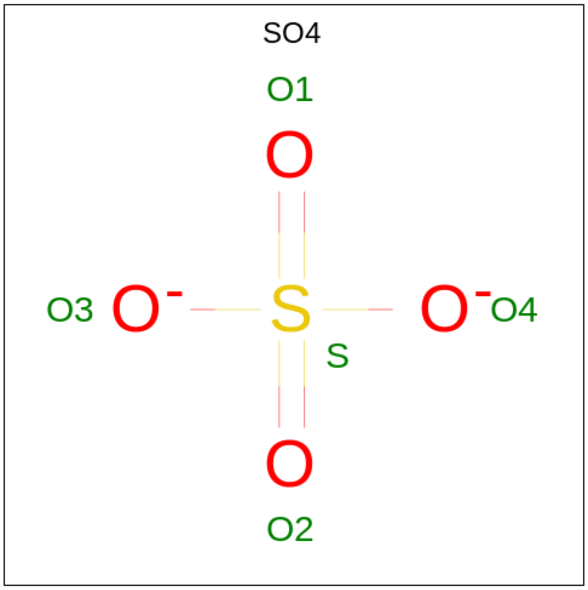

Mol	Chain	Residues	Atoms	ZeroOcc	AltConf
3	A	1	Total O S<br>5 4 1	0	0
3	A	1	Total O S<br>5 4 1	0	0

- Molecule 4 is water.

Mol	Chain	Residues	Atoms	ZeroOcc	AltConf
4	A	167	Total O<br>167 167	0	0
4	B	136	Total O<br>136 136	0	0

CONFIDENTIAL VALIDATION REPORT

3 Residue-property plots (i)

These plots are drawn for all protein, RNA, DNA and oligosaccharide chains in the entry. The first graphic for a chain summarises the proportions of the various outlier classes displayed in the second graphic. The second graphic shows the sequence view annotated by issues in geometry and electron density. Residues are color-coded according to the number of geometric quality criteria for which they contain at least one outlier: green = 0, yellow = 1, orange = 2 and red = 3 or more. A red dot above a residue indicates a poor fit to the electron density (RSRZ > 2). Stretches of 2 or more consecutive residues without any outlier are shown as a green connector. Residues present in the sample, but not in the model, are shown in grey.

- Molecule 1: Proto-oncogene tyrosine-protein kinase Src

Chain A:

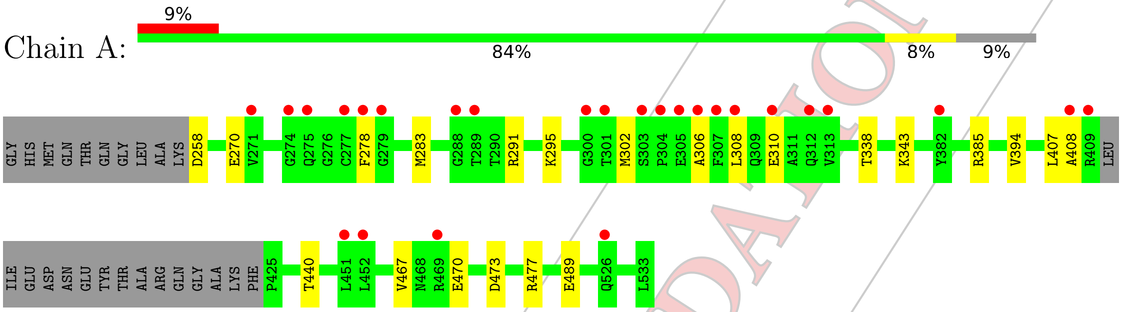

- Molecule 1: Proto-oncogene tyrosine-protein kinase Src

Chain B:

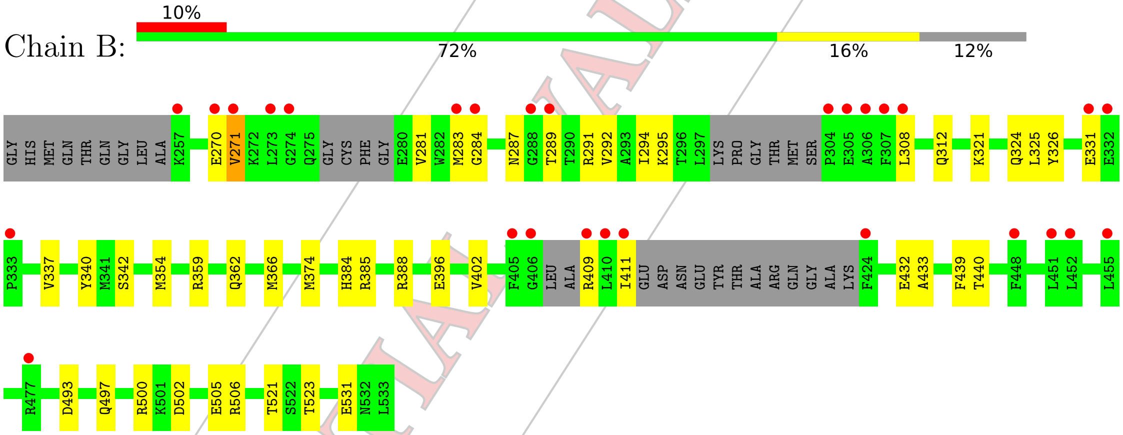

CONFIDENTIAL VALIDATION REPORT

4 Data and refinement statistics (i)

Property	Value	Source
Space group	P 1	Depositor
Cell constants<br>a, b, c, α, β, γ	41.80Å 62.96Å 74.14Å<br>78.98° 89.78° 89.69°	Depositor
Resolution (Å)	27.41 – 2.10<br>27.40 – 2.10	Depositor<br>EDS
% Data completeness<br>(in resolution range)	82.1 (27.41-2.10)<br>82.1 (27.40-2.10)	Depositor<br>EDS
R_{merge}	(Not available)	Depositor
R_{sym}	(Not available)	Depositor
$< I/\sigma(I) >$ [1]	1.52 (at 2.10Å)	Xtriage
Refinement program	PHENIX 1.11.1_2575	Depositor
R, R_{free}	0.185 , 0.222<br>0.188 , 0.218	Depositor<br>DCC
R_{free} test set	1000 reflections (2.83%)	wwPDB-VP
Wilson B-factor (Å^2)	28.7	Xtriage
Anisotropy	0.050	Xtriage
Bulk solvent k_{sol}(e/Å^3), B_{sol}(Å^2)	0.34 , 54.0	EDS
L-test for twinning[2]	$< \|L\| > = 0.51$, $< L^2 > = 0.34$	Xtriage
Estimated twinning fraction	0.020 for h,-k,-l	Xtriage
F_o,F_c correlation	0.94	EDS
Total number of atoms	4477	wwPDB-VP
Average B, all atoms (Å^2)	42.0	wwPDB-VP

Xtriage's analysis on translational NCS is as follows: *The largest off-origin peak in the Patterson function is 6.41% of the height of the origin peak. No significant pseudotranslation is detected.*

[1] Intensities estimated from amplitudes.

[2] Theoretical values of $< |L| >$, $< L^2 >$ for acentric reflections are 0.5, 0.333 respectively for untwinned datasets, and 0.375, 0.2 for perfectly twinned datasets.

CONFIDENTIAL VALIDATION REPORT

5 Model quality (i)

5.1 Standard geometry (i)

Bond lengths and bond angles in the following residue types are not validated in this section: 37O, SO4

The Z score for a bond length (or angle) is the number of standard deviations the observed value is removed from the expected value. A bond length (or angle) with $|Z| > 5$ is considered an outlier worth inspection. RMSZ is the root-mean-square of all Z scores of the bond lengths (or angles).

Mol	Chain	Bond lengths		Bond angles	
		RMSZ	#|Z| >5	RMSZ	#|Z| >5
1	A	0.33	0/2109	0.50	0/2862
1	B	0.35	0/2059	0.58	2/2789 (0.1%)
All	All	0.34	0/4168	0.54	2/5651 (0.0%)

There are no bond length outliers.

All (2) bond angle outliers are listed below:

Mol	Chain	Res	Type	Atoms	Z	Observed(°)	Ideal(°)
1	B	331	GLU	CB-CA-C	7.91	126.21	110.40
1	B	331	GLU	N-CA-C	-6.03	94.72	111.00

There are no chirality outliers.

There are no planarity outliers.

5.2 Too-close contacts (i)

In the following table, the Non-H and H(model) columns list the number of non-hydrogen atoms and hydrogen atoms in the chain respectively. The H(added) column lists the number of hydrogen atoms added and optimized by MolProbity. The Clashes column lists the number of clashes within the asymmetric unit, whereas Symm-Clashes lists symmetry-related clashes.

Mol	Chain	Non-H	H(model)	H(added)	Clashes	Symm-Clashes
1	A	2058	0	2008	20	0
1	B	2012	0	1972	33	0
2	A	47	0	39	3	0
2	B	47	0	40	1	0
3	A	10	0	0	0	0
4	A	167	0	0	6	1
4	B	136	0	0	4	1

Continued on next page...

CONFIDENTIAL VALIDATION REPORT

Continued from previous page...

Mol	Chain	Non-H	H(model)	H(added)	Clashes	Symm-Clashes
All	All	4477	0	4059	56	1

The all-atom clashscore is defined as the number of clashes found per 1000 atoms (including hydrogen atoms). The all-atom clashscore for this structure is 7.

All (56) close contacts within the same asymmetric unit are listed below, sorted by their clash magnitude.

Atom-1	Atom-2	Interatomic distance (Å)	Clash overlap (Å)
1:B:270:GLU:OE1	1:B:284:GLY:HA2	1.51	1.10
1:B:291:ARG:HH11	1:B:291:ARG:HG3	1.34	0.92
1:A:489:GLU:OE1	4:A:701:HOH:O	1.93	0.85
1:B:354:MET:SD	4:B:806:HOH:O	2.35	0.83
1:B:388:ARG:NH2	4:B:701:HOH:O	2.14	0.79
1:B:374:MET:HE3	1:B:402:VAL:HG11	1.67	0.76
1:B:359:ARG:NH2	1:B:531:GLU:O	2.22	0.73
1:A:343:LYS:O	4:A:703:HOH:O	2.06	0.72
1:B:291:ARG:HG3	1:B:291:ARG:NH1	2.02	0.70
1:A:283:MET:HG2	1:A:291:ARG:NH1	2.07	0.69
1:A:308:LEU:O	4:A:704:HOH:O	2.10	0.68
1:B:500:ARG:HD3	1:B:505:GLU:HB3	1.78	0.65
1:B:287:ASN:HB2	1:B:289:THR:HG22	1.79	0.64
1:A:278:PHE:CE2	1:A:295:LYS:HG2	2.34	0.62
1:B:493:ASP:O	1:B:497:GLN:HG3	2.03	0.59
1:B:283:MET:HE2	1:B:291:ARG:HD3	1.85	0.58
1:B:521:THR:OG1	4:B:702:HOH:O	2.16	0.58
1:A:258:ASP:N	4:A:710:HOH:O	2.38	0.57
1:B:384:HIS:O	1:B:385:ARG:HB2	2.04	0.57
1:B:292:VAL:CG1	1:B:337:VAL:HG13	2.35	0.56
1:B:374:MET:CE	1:B:402:VAL:HG11	2.36	0.56
1:B:432:GLU:HG2	1:B:433:ALA:N	2.21	0.55
2:A:601:37O:N04	2:A:601:37O:H5	2.22	0.55
1:B:326:TYR:HB2	1:B:337:VAL:HG12	1.88	0.54
1:B:292:VAL:HG11	1:B:337:VAL:HG13	1.89	0.54
1:B:502:ASP:HB3	1:B:505:GLU:HG3	1.90	0.53
1:A:302:MET:HE1	1:A:407:LEU:HD11	1.91	0.53
2:B:601:37O:H5	2:B:601:37O:N04	2.24	0.52
1:B:271:VAL:HG22	1:B:283:MET:HB3	1.89	0.52
1:B:432:GLU:OE2	1:B:506:ARG:NH2	2.42	0.52
1:A:291:ARG:HG3	1:A:291:ARG:HH11	1.74	0.51
1:B:281:VAL:HG22	1:B:295:LYS:HG3	1.93	0.50
1:B:523:THR:O	4:B:703:HOH:O	2.20	0.50

Continued on next page...

CONFIDENTIAL VALIDATION REPORT

Continued from previous page...

Atom-1	Atom-2	Interatomic distance (Å)	Clash overlap (Å)
1:A:270:GLU:OE1	1:A:291:ARG:NH2	2.40	0.49
1:A:306:ALA:O	1:A:310:GLU:HG2	2.12	0.49
1:A:278:PHE:CD1	1:A:408:ALA:HB2	2.48	0.48
1:A:467:VAL:HG22	1:A:470:GLU:HB2	1.96	0.48
1:A:283:MET:HG2	1:A:291:ARG:HH11	1.77	0.48
1:B:385:ARG:HH21	1:B:409:ARG:N	2.12	0.47
1:A:338:THR:HG23	2:A:601:37O:H21	1.96	0.47
1:A:473:ASP:O	1:A:477:ARG:HG3	2.14	0.47
1:B:411:ILE:HD11	1:B:440:THR:HG22	1.96	0.46
1:B:385:ARG:HG2	1:B:439:PHE:CD2	2.50	0.46
1:B:362:GLN:O	1:B:366:MET:HG3	2.16	0.46
1:A:440:THR:OG1	4:A:702:HOH:O	1.94	0.46
1:A:385:ARG:NH1	4:A:712:HOH:O	2.40	0.45
1:B:374:MET:HE3	1:B:402:VAL:HG21	1.99	0.44
1:B:324:GLN:NE2	1:B:325:LEU:O	2.47	0.44
1:B:321:LYS:HA	1:B:321:LYS:HD3	1.79	0.43
1:A:467:VAL:HG23	1:A:470:GLU:H	1.83	0.43
1:A:291:ARG:NH1	1:A:291:ARG:HG3	2.32	0.43
1:B:308:LEU:O	1:B:312:GLN:HG2	2.17	0.43
1:B:292:VAL:HG12	1:B:294:ILE:HG13	2.02	0.41
1:A:343:LYS:HB2	1:A:394:VAL:HB	2.02	0.41
2:A:601:37O:H17	2:A:601:37O:H24	1.69	0.41
1:B:340:TYR:CE2	1:B:342:SER:HA	2.55	0.40

All (1) symmetry-related close contacts are listed below. The label for Atom-2 includes the symmetry operator and encoded unit-cell translations to be applied.

Atom-1	Atom-2	Interatomic distance (Å)	Clash overlap (Å)
4:A:841:HOH:O	4:B:717:HOH:O[1_545]	2.09	0.11

5.3 Torsion angles (i)

5.3.1 Protein backbone (i)

In the following table, the Percentiles column shows the percent Ramachandran outliers of the chain as a percentile score with respect to all X-ray entries followed by that with respect to entries of similar resolution.

The Analysed column shows the number of residues for which the backbone conformation was analysed, and the total number of residues.

CONFIDENTIAL VALIDATION REPORT

Mol	Chain	Analysed	Favoured	Allowed	Outliers	Percentiles	
1	A	257/286 (90%)	253 (98%)	4 (2%)	0	100	100
1	B	243/286 (85%)	235 (97%)	8 (3%)	0	100	100
All	All	500/572 (87%)	488 (98%)	12 (2%)	0	100	100

There are no Ramachandran outliers to report.

5.3.2 Protein sidechains (i)

In the following table, the Percentiles column shows the percent sidechain outliers of the chain as a percentile score with respect to all X-ray entries followed by that with respect to entries of similar resolution.

The Analysed column shows the number of residues for which the sidechain conformation was analysed, and the total number of residues.

Mol	Chain	Analysed	Rotameric	Outliers	Percentiles	
1	A	216/245 (88%)	216 (100%)	0	100	100
1	B	212/245 (86%)	210 (99%)	2 (1%)	78	84
All	All	428/490 (87%)	426 (100%)	2 (0%)	88	92

All (2) residues with a non-rotameric sidechain are listed below:

Mol	Chain	Res	Type
1	B	271	VAL
1	B	396	GLU

Sometimes sidechains can be flipped to improve hydrogen bonding and reduce clashes. There are no such sidechains identified.

5.3.3 RNA (i)

There are no RNA molecules in this entry.

5.4 Non-standard residues in protein, DNA, RNA chains (i)

There are no non-standard protein/DNA/RNA residues in this entry.

CONFIDENTIAL VALIDATION REPORT

5.5 Carbohydrates (i)

There are no monosaccharides in this entry.

5.6 Ligand geometry (i)

4 ligands are modelled in this entry.

In the following table, the Counts columns list the number of bonds (or angles) for which Mogul statistics could be retrieved, the number of bonds (or angles) that are observed in the model and the number of bonds (or angles) that are defined in the Chemical Component Dictionary. The Link column lists molecule types, if any, to which the group is linked. The Z score for a bond length (or angle) is the number of standard deviations the observed value is removed from the expected value. A bond length (or angle) with $|Z| > 2$ is considered an outlier worth inspection. RMSZ is the root-mean-square of all Z scores of the bond lengths (or angles).

Mol	Type	Chain	Res	Link	Bond lengths			Bond angles		
					Counts	RMSZ	$\#\|Z\| > 2$	Counts	RMSZ	$\#\|Z\| > 2$
2	37O	A	601	1	52,52,52	1.60	9 (17%)	66,73,73	1.97	17 (25%)
2	37O	B	601	-	52,52,52	1.75	8 (15%)	66,73,73	2.29	15 (22%)
3	SO4	A	603	-	4,4,4	0.15	0	6,6,6	0.06	0
3	SO4	A	602	-	4,4,4	0.14	0	6,6,6	0.05	0

In the following table, the Chirals column lists the number of chiral outliers, the number of chiral centers analysed, the number of these observed in the model and the number defined in the Chemical Component Dictionary. Similar counts are reported in the Torsion and Rings columns. '-' means no outliers of that kind were identified.

Mol	Type	Chain	Res	Link	Chirals	Torsions	Rings
2	37O	A	601	1	-	4/26/52/52	0/6/6/6
2	37O	B	601	-	-	4/26/52/52	0/6/6/6

All (17) bond length outliers are listed below:

Mol	Chain	Res	Type	Atoms	Z	Observed(Å)	Ideal(Å)
2	B	601	37O	C36-C37	-6.39	1.40	1.51
2	B	601	37O	C10-C06	-6.08	1.37	1.51
2	A	601	37O	C10-C06	-5.26	1.39	1.51
2	A	601	37O	C36-C37	-4.78	1.42	1.51
2	B	601	37O	C25-N09	-4.29	1.34	1.43
2	A	601	37O	C25-N09	-3.90	1.35	1.43
2	B	601	37O	C40-N43	-3.44	1.34	1.41
2	B	601	37O	C05-N07	-3.17	1.34	1.39

Continued on next page...

CONFIDENTIAL VALIDATION REPORT

Continued from previous page...

Mol	Chain	Res	Type	Atoms	Z	Observed(Å)	Ideal(Å)
2	A	601	37O	C40-N43	-2.95	1.35	1.41
2	A	601	37O	C36-N07	2.81	1.51	1.46
2	A	601	37O	C05-N07	-2.75	1.35	1.39
2	B	601	37O	C12-N11	-2.65	1.34	1.40
2	A	601	37O	C12-N11	-2.60	1.35	1.40
2	A	601	37O	C01-N02	2.52	1.39	1.34
2	A	601	37O	C08-N09	-2.45	1.34	1.37
2	B	601	37O	C01-N02	2.42	1.39	1.34
2	B	601	37O	C36-N07	2.36	1.50	1.46

All (32) bond angle outliers are listed below:

Mol	Chain	Res	Type	Atoms	Z	Observed(^{o})	Ideal(^{o})
2	B	601	37O	C36-N07-C08	10.05	123.05	116.77
2	A	601	37O	N02-C03-N04	-7.01	119.91	126.55
2	B	601	37O	N02-C03-N04	-6.50	120.39	126.55
2	A	601	37O	C36-N07-C08	6.11	120.59	116.77
2	B	601	37O	C40-N43-C44	-5.94	117.10	127.50
2	B	601	37O	C37-C36-N07	4.73	121.36	113.39
2	A	601	37O	C26-C25-N09	4.55	126.37	119.85
2	B	601	37O	C23-N18-C19	3.96	120.26	111.52
2	B	601	37O	C10-N09-C25	-3.92	112.62	117.98
2	A	601	37O	C30-C25-N09	-3.52	114.80	119.85
2	A	601	37O	C34-O32-C27	-3.42	110.09	117.51
2	B	601	37O	C06-C01-N02	-3.30	118.31	123.82
2	B	601	37O	C01-C06-C05	3.30	120.12	115.66
2	A	601	37O	C05-N07-C08	-3.25	119.16	122.22
2	A	601	37O	O35-C08-N09	-3.20	117.90	122.22
2	B	601	37O	C01-N02-C03	2.97	120.36	115.88
2	A	601	37O	C01-N02-C03	2.89	120.25	115.88
2	A	601	37O	C06-C01-N02	-2.85	119.07	123.82
2	A	601	37O	C33-O31-C29	-2.71	111.63	117.51
2	A	601	37O	C01-C06-C05	2.68	119.28	115.66
2	B	601	37O	C05-N07-C08	-2.58	119.78	122.22
2	A	601	37O	C23-N18-C19	2.54	117.13	111.52
2	A	601	37O	C40-N43-C44	-2.51	123.11	127.50
2	B	601	37O	C06-C10-N09	2.45	113.55	109.76
2	B	601	37O	C16-C15-N18	-2.41	118.06	121.38
2	A	601	37O	C22-N21-C20	2.40	112.88	109.52
2	A	601	37O	C03-N04-C05	2.39	122.60	114.34
2	A	601	37O	N11-C03-N04	2.33	124.82	116.92
2	B	601	37O	N11-C03-N04	2.31	124.75	116.92

Continued on next page...

CONFIDENTIAL VALIDATION REPORT

Continued from previous page...

Mol	Chain	Res	Type	Atoms	Z	Observed(^{o})	Ideal(^{o})
2	B	601	37O	C03-N04-C05	2.23	122.03	114.34
2	A	601	37O	C06-C10-N09	2.18	113.13	109.76
2	B	601	37O	C30-C25-N09	2.16	122.94	119.85

There are no chirality outliers.

All (8) torsion outliers are listed below:

Mol	Chain	Res	Type	Atoms
2	B	601	37O	C41-C40-N43-C44
2	B	601	37O	C39-C40-N43-C44
2	A	601	37O	C26-C27-O32-C34
2	A	601	37O	C28-C27-O32-C34
2	A	601	37O	C37-C36-N07-C05
2	B	601	37O	O47-C44-C45-C46
2	A	601	37O	C37-C36-N07-C08
2	B	601	37O	N43-C44-C45-C46

There are no ring outliers.

2 monomers are involved in 4 short contacts:

Mol	Chain	Res	Type	Clashes	Symm-Clashes
2	A	601	37O	3	0
2	B	601	37O	1	0

The following is a two-dimensional graphical depiction of Mogul quality analysis of bond lengths, bond angles, torsion angles, and ring geometry for all instances of the Ligand of Interest. In addition, ligands with molecular weight > 250 and outliers as shown on the validation Tables will also be included. For torsion angles, if less then 5% of the Mogul distribution of torsion angles is within 10 degrees of the torsion angle in question, then that torsion angle is considered an outlier. Any bond that is central to one or more torsion angles identified as an outlier by Mogul will be highlighted in the graph. For rings, the root-mean-square deviation (RMSD) between the ring in question and similar rings identified by Mogul is calculated over all ring torsion angles. If the average RMSD is greater than 60 degrees and the minimal RMSD between the ring in question and any Mogul-identified rings is also greater than 60 degrees, then that ring is considered an outlier. The outliers are highlighted in purple. The color gray indicates Mogul did not find sufficient equivalents in the CSD to analyse the geometry.

CONFIDENTIAL VALIDATION REPORT

Ligand 37O A 601

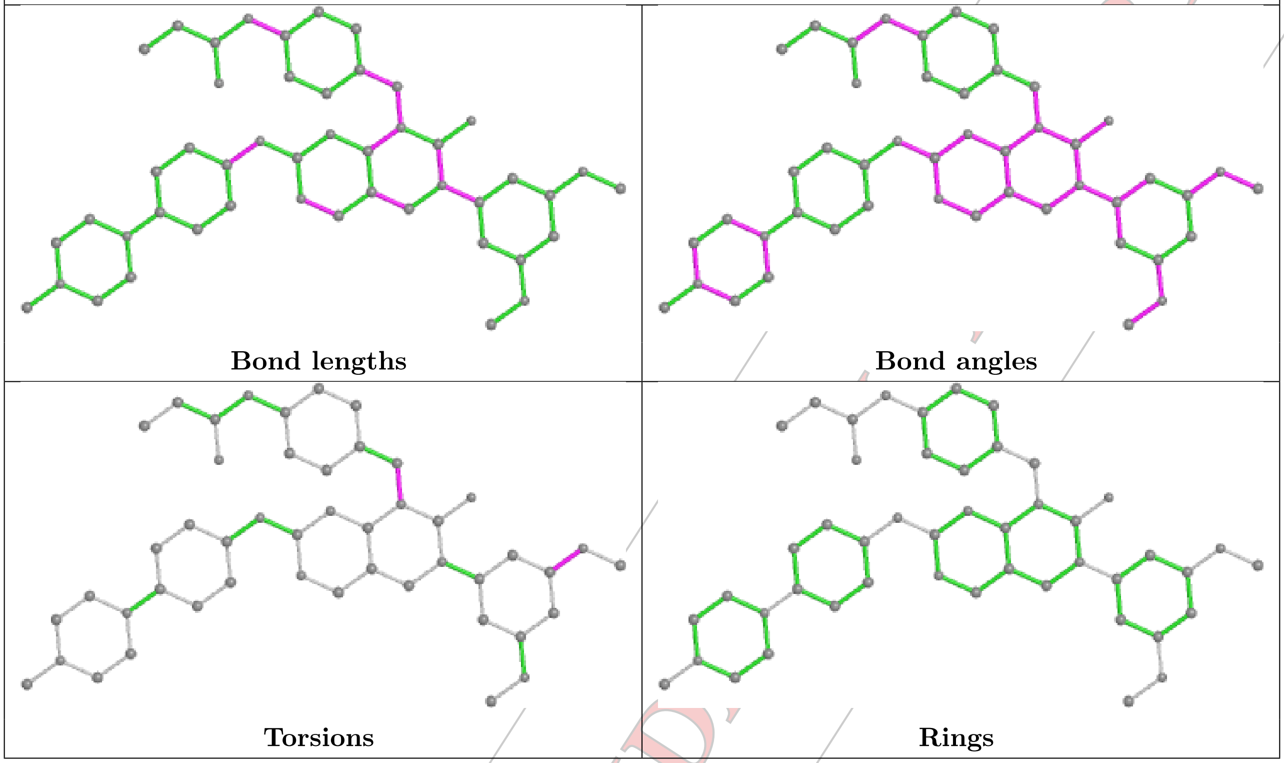

Ligand 37O B 601

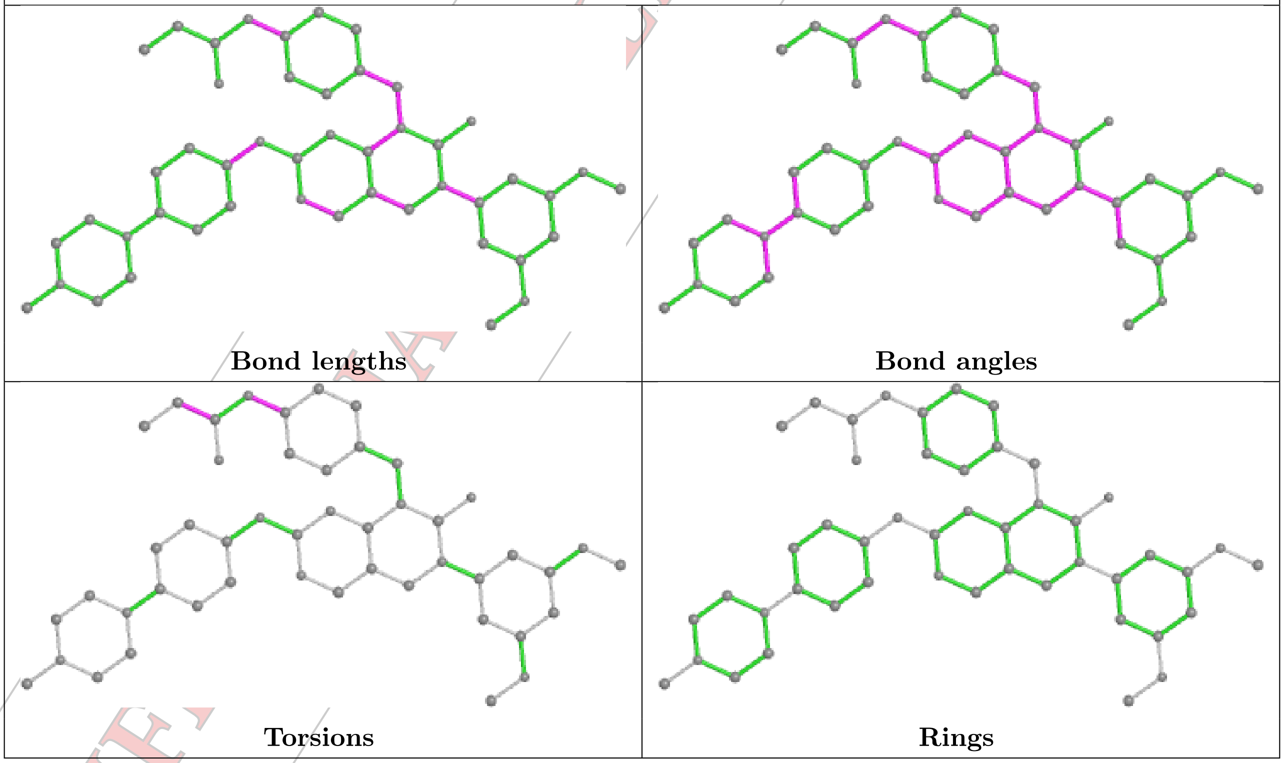

5.7 Other polymers (i)

There are no such residues in this entry.

5.8 Polymer linkage issues (i)

There are no chain breaks in this entry.

CONFIDENTIAL VALIDATION REPORT

6 Fit of model and data (i)

6.1 Protein, DNA and RNA chains (i)

In the following table, the column labelled '#RSRZ> 2' contains the number (and percentage) of RSRZ outliers, followed by percent RSRZ outliers for the chain as percentile scores relative to all X-ray entries and entries of similar resolution. The OWAB column contains the minimum, median, 95th percentile and maximum values of the occupancy-weighted average B-factor per residue. The column labelled 'Q< 0.9' lists the number of (and percentage) of residues with an average occupancy less than 0.9.

Mol	Chain	Analysed	<RSRZ>	#RSRZ>2			OWAB(Å^2)	Q<0.9
1	A	261/286 (91%)	0.47	26 (9%)	7	9	17, 37, 75, 88	0
1	B	253/286 (88%)	0.56	28 (11%)	5	7	18, 38, 85, 100	0
All	All	514/572 (89%)	0.51	54 (10%)	6	8	17, 38, 80, 100	0

All (54) RSRZ outliers are listed below:

Mol	Chain	Res	Type	RSRZ
1	B	424	PHE	7.5
1	B	306	ALA	7.2
1	B	411	ILE	6.6
1	A	300	GLY	6.3
1	B	307	PHE	5.8
1	A	306	ALA	5.5
1	A	303	SER	4.7
1	B	289	THR	4.5
1	A	308	LEU	4.4
1	A	304	PRO	4.3
1	B	288	GLY	4.3
1	A	275	GLN	4.1
1	B	410	LEU	4.1
1	B	271	VAL	3.9
1	A	271	VAL	3.6
1	A	305	GLU	3.6
1	A	301	THR	3.6
1	A	526	GLN	3.5
1	B	308	LEU	3.5
1	B	274	GLY	3.3
1	A	409	ARG	3.2
1	B	270	GLU	3.1
1	B	304	PRO	3.1
1	B	406	GLY	3.0

Continued on next page...

CONFIDENTIAL VALIDATION REPORT

Continued from previous page...

Mol	Chain	Res	Type	RSRZ
1	A	277	CYS	3.0
1	A	451	LEU	3.0
1	A	274	GLY	2.9
1	B	305	GLU	2.9
1	B	451	LEU	2.9
1	B	283	MET	2.7
1	A	289	THR	2.7
1	A	307	PHE	2.7
1	A	310	GLU	2.7
1	B	257	LYS	2.5
1	A	469	ARG	2.5
1	B	332	GLU	2.5
1	B	333	PRO	2.4
1	B	273	LEU	2.4
1	A	452	LEU	2.3
1	B	409	ARG	2.3
1	A	278	PHE	2.3
1	B	448	PHE	2.2
1	B	455	LEU	2.2
1	A	382	TYR	2.2
1	A	313	VAL	2.2
1	B	405	PHE	2.2
1	A	279	GLY	2.1
1	B	477	ARG	2.1
1	B	452	LEU	2.1
1	A	408	ALA	2.0
1	A	288	GLY	2.0
1	B	331	GLU	2.0
1	B	284	GLY	2.0
1	A	312	GLN	2.0

6.2 Non-standard residues in protein, DNA, RNA chains (i)

There are no non-standard protein/DNA/RNA residues in this entry.

6.3 Carbohydrates (i)

There are no monosaccharides in this entry.

CONFIDENTIAL VALIDATION REPORT

6.4 Ligands (i)

In the following table, the Atoms column lists the number of modelled atoms in the group and the number defined in the chemical component dictionary. The B-factors column lists the minimum, median, 95th percentile and maximum values of B factors of atoms in the group. The column labelled 'Q< 0.9' lists the number of atoms with occupancy less than 0.9.

Mol	Type	Chain	Res	Atoms	RSCC	RSR	B-factors($Å^2$)	Q<0.9
3	SO4	A	603	5/5	0.76	0.34	138,139,139,140	0
2	37O	B	601	47/47	0.87	0.17	48,54,74,76	0
3	SO4	A	602	5/5	0.89	0.41	142,142,142,143	0
2	37O	A	601	47/47	0.89	0.15	26,42,58,61	0

The following is a graphical depiction of the model fit to experimental electron density of all instances of the Ligand of Interest. In addition, ligands with molecular weight > 250 and outliers as shown on the geometry validation Tables will also be included. Each fit is shown from different orientation to approximate a three-dimensional view.

Electron density around 37O B 601:

2mF$_o$-DF$_c$ (at 0.7 rmsd) in gray
mF$_o$-DF$_c$ (at 3 rmsd) in purple (negative)
and green (positive)

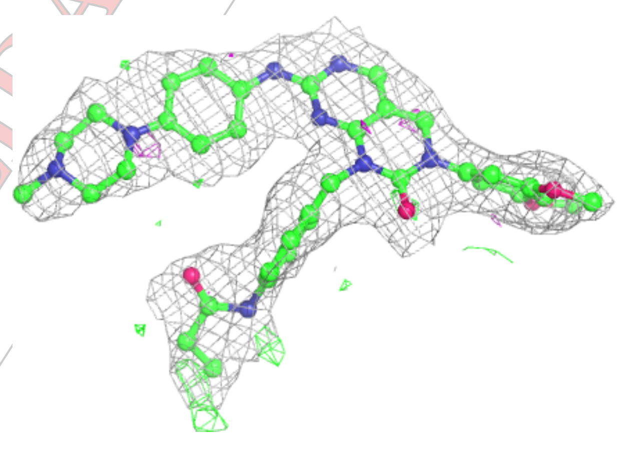

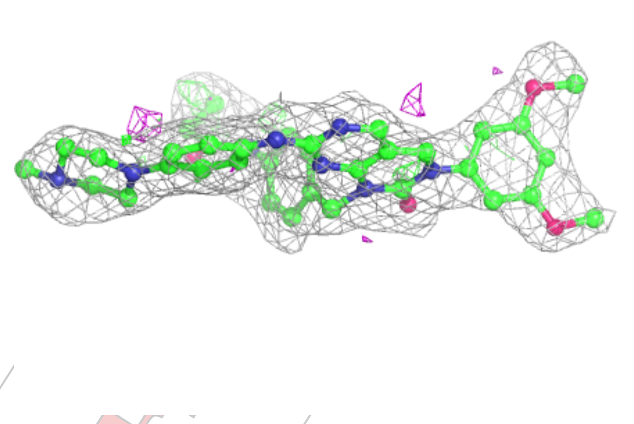

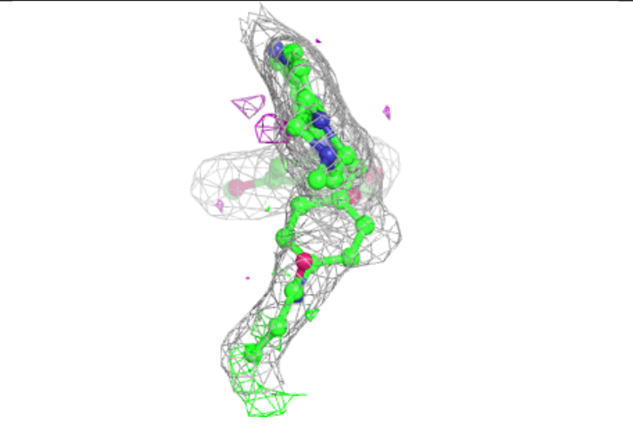

CONFIDENTIAL VALIDATION REPORT

Electron density around 37O A 601:

2mF$_o$-DF$_c$ (at 0.7 rmsd) in gray
mF$_o$-DF$_c$ (at 3 rmsd) in purple (negative)
and green (positive)

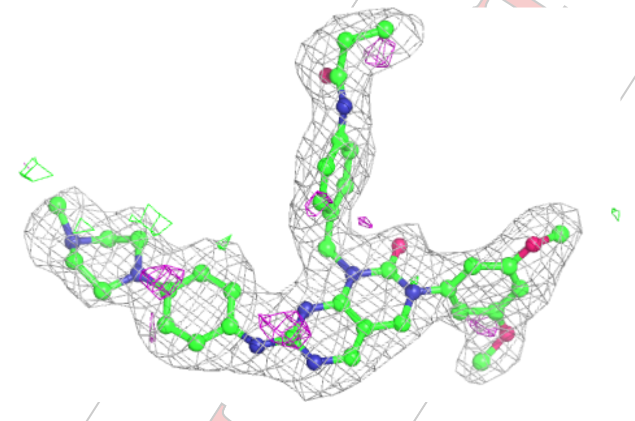

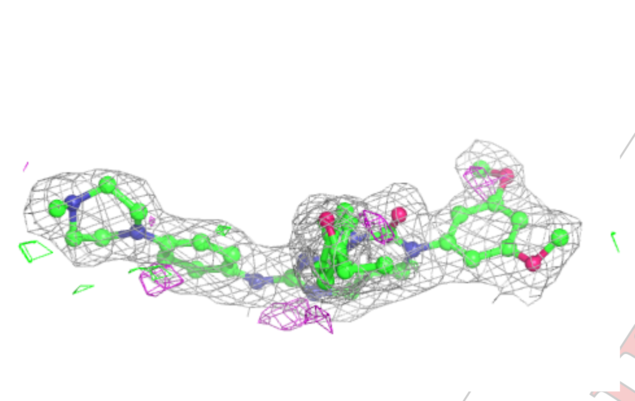

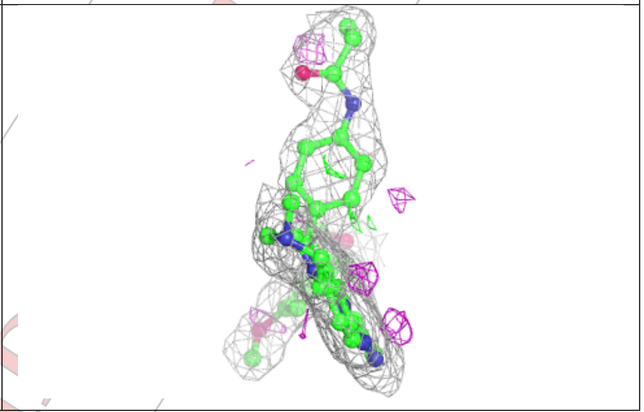

6.5 Other polymers (i)

There are no such residues in this entry.

CONFIDENTIAL
