# Supplementary material for: Structural insights into the potency and selectivity of covalent pan-FGFR inhibitors
Source: Commun Chem. 2022 Jan 11;5:5. doi: 10.1038/s42004-021-00623-x (PMC9814232; doi:10.1038/s42004-021-00623-x)
Supplement: Supplementary file 4 — Supplementary Data 1 [file 42004_2021_623_MOESM4_ESM.zip › validation report/D_1300018774_val-report-full_P1.pdf (1).V11]

Full wwPDB X-ray Structure Validation Report (i)

Aug 11, 2021 – 08:25 PM JST

PDB ID	:	7D5O
Title	:	C-Src in complex with TAS-120
Deposited on	:	2020-09-27
Resolution	:	2.69 Å(reported)

This is a Full wwPDB X-ray Structure Validation Report.

This report is produced by the wwPDB biocuration pipeline after annotation of the structure.

We welcome your comments at *validation@mail.wwpdb.org*
A user guide is available at
https://www.wwpdb.org/validation/2017/XrayValidationReportHelp
with specific help available everywhere you see the (i) symbol.

The following versions of software and data (see references (i)) were used in the production of this report:

MolProbity	:	4.02b-467
Mogul	:	1.8.5 (274361), CSD as541be (2020)
Xtriage (Phenix)	:	1.13
EDS	:	2.23.1
buster-report	:	1.1.7 (2018)
Percentile statistics	:	20191225.v01 (using entries in the PDB archive December 25th 2019)
Refmac	:	5.8.0158
CCP4	:	7.0.044 (Gargrove)
Ideal geometry (proteins)	:	Engh & Huber (2001)
Ideal geometry (DNA, RNA)	:	Parkinson et al. (1996)
Validation Pipeline (wwPDB-VP)	:	2.23.1

CONFIDENTIAL VALIDATION REPORT

1 Overall quality at a glance (i)

The following experimental techniques were used to determine the structure:
X-RAY DIFFRACTION

The reported resolution of this entry is 2.69 Å.

Percentile scores (ranging between 0-100) for global validation metrics of the entry are shown in the following graphic. The table shows the number of entries on which the scores are based.

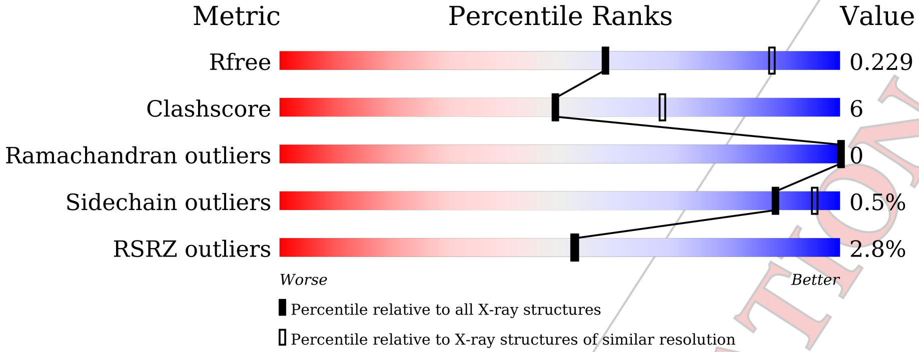

Metric	Whole archive (#Entries)	Similar resolution (#Entries, resolution range(Å))
R_{free}	130704	3863 (2.70-2.66)
Clashscore	141614	4210 (2.70-2.66)
Ramachandran outliers	138981	4141 (2.70-2.66)
Sidechain outliers	138945	4141 (2.70-2.66)
RSRZ outliers	127900	3780 (2.70-2.66)

The table below summarises the geometric issues observed across the polymeric chains and their fit to the electron density. The red, orange, yellow and green segments of the lower bar indicate the fraction of residues that contain outliers for >=3, 2, 1 and 0 types of geometric quality criteria respectively. A grey segment represents the fraction of residues that are not modelled. The numeric value for each fraction is indicated below the corresponding segment, with a dot representing fractions <=5% The upper red bar (where present) indicates the fraction of residues that have poor fit to the electron density. The numeric value is given above the bar.

Mol	Chain	Length	Quality of chain
1	A	286	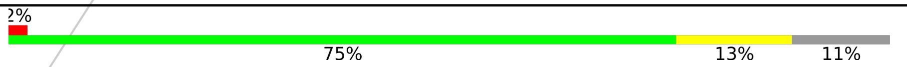 2% 75% 13% 11%
1	B	286	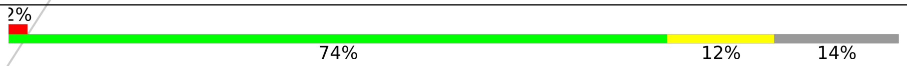 2% 74% 12% 14%

CONFIDENTIAL VALIDATION REPORT

2 Entry composition (i)

There are 3 unique types of molecules in this entry. The entry contains 4092 atoms, of which 0 are hydrogens and 0 are deuteriums.

In the tables below, the ZeroOcc column contains the number of atoms modelled with zero occupancy, the AltConf column contains the number of residues with at least one atom in alternate conformation and the Trace column contains the number of residues modelled with at most 2 atoms.

- Molecule 1 is a protein called Proto-oncogene tyrosine-protein kinase Src.

Mol	Chain	Residues	Atoms					ZeroOcc	AltConf	Trace
1	B	247	Total<br>1972	C<br>1268	N<br>327	O<br>361	S<br>16	0	0	0
1	A	254	Total<br>2002	C<br>1283	N<br>331	O<br>372	S<br>16	0	0	0

There are 6 discrepancies between the modelled and reference sequences:

Chain	Residue	Modelled	Actual	Comment	Reference
B	248	GLY	-	expression tag	UNP P00523
B	249	HIS	-	expression tag	UNP P00523
B	250	MET	-	expression tag	UNP P00523
A	248	GLY	-	expression tag	UNP P00523
A	249	HIS	-	expression tag	UNP P00523
A	250	MET	-	expression tag	UNP P00523

- Molecule 2 is Futibatinib (three-letter code: TAW) (formula: $C_{22}H_{22}N_6O_3$) (labeled as "Ligand of Interest" by depositor).

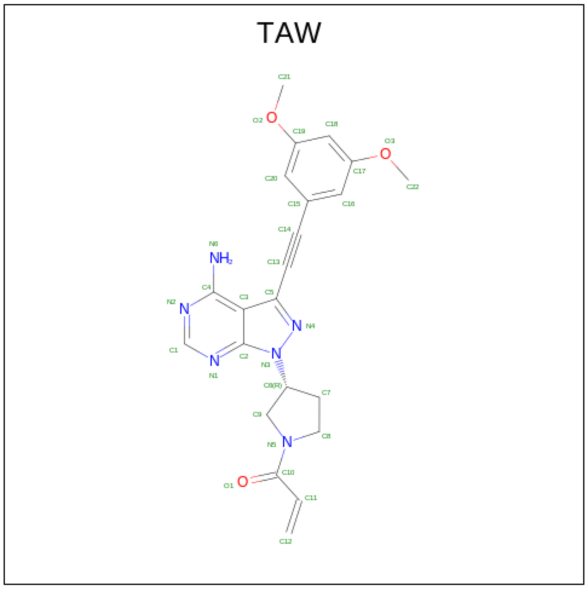

Mol	Chain	Residues	Atoms				ZeroOcc	AltConf
2	B	1	Total<br>31	C<br>22	N<br>6	O<br>3	0	0
2	A	1	Total<br>31	C<br>22	N<br>6	O<br>3	0	0

- Molecule 3 is water.

Mol	Chain	Residues	Atoms		ZeroOcc	AltConf
3	B	28	Total<br>28	O<br>28	0	0
3	A	28	Total<br>28	O<br>28	0	0

CONFIDENTIAL VALIDATION REPORT

3 Residue-property plots (i)

These plots are drawn for all protein, RNA, DNA and oligosaccharide chains in the entry. The first graphic for a chain summarises the proportions of the various outlier classes displayed in the second graphic. The second graphic shows the sequence view annotated by issues in geometry and electron density. Residues are color-coded according to the number of geometric quality criteria for which they contain at least one outlier: green = 0, yellow = 1, orange = 2 and red = 3 or more. A red dot above a residue indicates a poor fit to the electron density (RSRZ > 2). Stretches of 2 or more consecutive residues without any outlier are shown as a green connector. Residues present in the sample, but not in the model, are shown in grey.

- Molecule 1: Proto-oncogene tyrosine-protein kinase Src

Chain B:

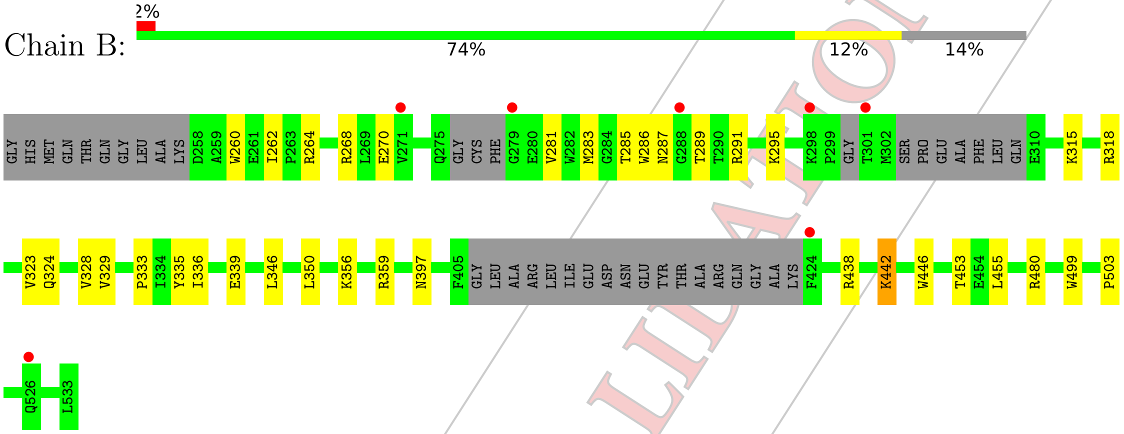

- Molecule 1: Proto-oncogene tyrosine-protein kinase Src

Chain A:

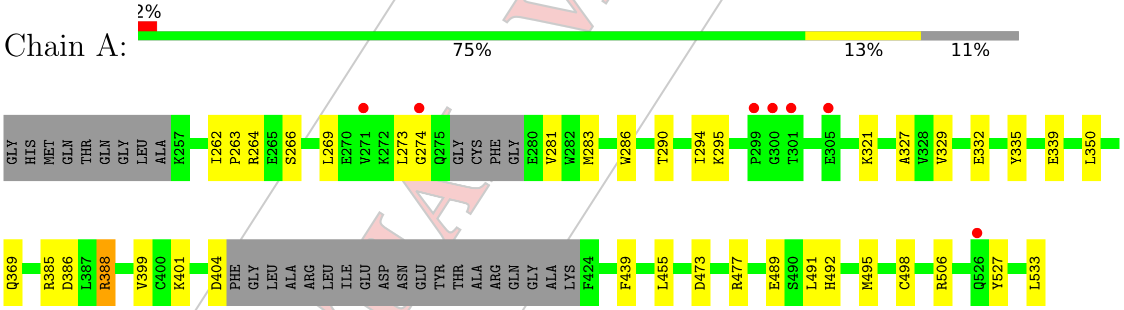

CONFIDENTIAL VALIDATION REPORT

4 Data and refinement statistics (i)

Property	Value	Source
Space group	P 1	Depositor
Cell constants<br>a, b, c, α, β, γ	41.97Å 63.18Å 73.14Å<br>100.64° 90.61° 89.82°	Depositor
Resolution (Å)	28.88 – 2.69<br>28.88 – 2.69	Depositor<br>EDS
% Data completeness<br>(in resolution range)	96.1 (28.88-2.69)<br>96.1 (28.88-2.69)	Depositor<br>EDS
R_{merge}	(Not available)	Depositor
R_{sym}	(Not available)	Depositor
$< I/\sigma(I) >$ [1]	2.05 (at 2.68Å)	Xtriage
Refinement program	PHENIX 1.11.1_2575	Depositor
R, R_{free}	0.179 , 0.231<br>0.178 , 0.229	Depositor<br>DCC
R_{free} test set	1979 reflections (9.98%)	wwPDB-VP
Wilson B-factor (Å^2)	46.1	Xtriage
Anisotropy	0.049	Xtriage
Bulk solvent k_{sol}(e/Å^3), B_{sol}(Å^2)	0.33 , 45.1	EDS
L-test for twinning[2]	$< \|L\| >$ = 0.50, $< L^2 >$ = 0.33	Xtriage
Estimated twinning fraction	0.024 for h,-k,-l	Xtriage
F_o,F_c correlation	0.94	EDS
Total number of atoms	4092	wwPDB-VP
Average B, all atoms (Å^2)	53.0	wwPDB-VP

Xtriage's analysis on translational NCS is as follows: *The largest off-origin peak in the Patterson function is 7.30% of the height of the origin peak. No significant pseudotranslation is detected.*

[1] Intensities estimated from amplitudes.

[2] Theoretical values of $< |L| >$, $< L^2 >$ for acentric reflections are 0.5, 0.333 respectively for untwinned datasets, and 0.375, 0.2 for perfectly twinned datasets.

CONFIDENTIAL VALIDATION REPORT

5 Model quality (i)

5.1 Standard geometry (i)

Bond lengths and bond angles in the following residue types are not validated in this section: TAW

The Z score for a bond length (or angle) is the number of standard deviations the observed value is removed from the expected value. A bond length (or angle) with $|Z| > 5$ is considered an outlier worth inspection. RMSZ is the root-mean-square of all Z scores of the bond lengths (or angles).

Mol	Chain	Bond lengths		Bond angles	
		RMSZ	#|Z| >5	RMSZ	#|Z| >5
1	A	0.33	0/2050	0.47	0/2785
1	B	0.29	0/2018	0.45	0/2734
All	All	0.31	0/4068	0.46	0/5519

Chiral center outliers are detected by calculating the chiral volume of a chiral center and verifying if the center is modelled as a planar moiety or with the opposite hand.A planarity outlier is detected by checking planarity of atoms in a peptide group, atoms in a mainchain group or atoms of a sidechain that are expected to be planar.

Mol	Chain	#Chirality outliers	#Planarity outliers
1	A	0	1

There are no bond length outliers.

There are no bond angle outliers.

There are no chirality outliers.

All (1) planarity outliers are listed below:

Mol	Chain	Res	Type	Group
1	A	388	ARG	Sidechain

5.2 Too-close contacts (i)

In the following table, the Non-H and H(model) columns list the number of non-hydrogen atoms and hydrogen atoms in the chain respectively. The H(added) column lists the number of hydrogen atoms added and optimized by MolProbity. The Clashes column lists the number of clashes within the asymmetric unit, whereas Symm-Clashes lists symmetry-related clashes.

CONFIDENTIAL VALIDATION REPORT

Mol	Chain	Non-H	H(model)	H(added)	Clashes	Symm-Clashes
1	A	2002	0	1943	26	0
1	B	1972	0	1935	21	1
2	A	31	0	0	1	0
2	B	31	0	0	0	0
3	A	28	0	0	0	0
3	B	28	0	0	1	0
All	All	4092	0	3878	47	1

The all-atom clashscore is defined as the number of clashes found per 1000 atoms (including hydrogen atoms). The all-atom clashscore for this structure is 6.

All (47) close contacts within the same asymmetric unit are listed below, sorted by their clash magnitude.

Atom-1	Atom-2	Interatomic distance (Å)	Clash overlap (Å)
1:A:321:LYS:NZ	1:A:369:GLN:HE21	1.63	0.95
1:A:321:LYS:HZ2	1:A:369:GLN:HE21	1.12	0.94
1:B:324:GLN:NE2	3:B:701:HOH:O	2.02	0.91
1:A:369:GLN:HE22	1:A:399:VAL:HA	1.36	0.88
1:B:323:VAL:HG23	1:B:339:GLU:HG2	1.61	0.81
1:A:286:TRP:HB3	1:A:290:THR:HG23	1.67	0.77
1:B:264:ARG:NH1	1:B:333:PRO:O	2.22	0.72
1:A:527:TYR:OH	1:A:533:LEU:O	2.13	0.66
1:B:287:ASN:HB2	1:B:289:THR:HG22	1.81	0.62
1:B:270:GLU:OE2	1:B:285:THR:OG1	2.18	0.61
1:A:473:ASP:O	1:A:477:ARG:HG2	2.02	0.60
1:A:269:LEU:HG	1:A:294:ILE:HD12	1.86	0.56
1:A:386:ASP:OD2	1:A:388:ARG:NH1	2.38	0.56
1:A:404:ASP:OD1	1:A:404:ASP:N	2.36	0.56
1:B:328:VAL:HG23	1:B:336:ILE:HD13	1.88	0.56
1:B:260:TRP:CD1	1:B:315:LYS:HD3	2.41	0.54
1:B:323:VAL:HG23	1:B:339:GLU:CG	2.33	0.54
1:A:386:ASP:OD1	1:A:388:ARG:NH1	2.40	0.52
1:B:350:LEU:HD11	1:B:455:LEU:HD23	1.92	0.51
1:B:318:ARG:HA	1:B:324:GLN:HE22	1.76	0.51
1:B:329:VAL:HG22	1:B:335:TYR:H	1.76	0.51
1:A:489:GLU:OE1	1:A:492:HIS:ND1	2.38	0.51
1:A:329:VAL:HB	1:A:335:TYR:HB2	1.92	0.51
1:B:262:ILE:HD11	1:B:286:TRP:HE1	1.77	0.50
1:A:339:GLU:OE1	1:A:401:LYS:NZ	2.46	0.49
1:B:442:LYS:HG3	1:B:503:PRO:O	2.13	0.48
1:A:498:CYS:O	1:A:506:ARG:HD3	2.15	0.47
1:A:385:ARG:HG2	1:A:439:PHE:CD1	2.49	0.47

Continued on next page...

CONFIDENTIAL VALIDATION REPORT

Continued from previous page...

Atom-1	Atom-2	Interatomic distance (Å)	Clash overlap (Å)
1:B:346:LEU:HD21	1:B:455:LEU:HD21	1.97	0.46
1:A:350:LEU:HD11	1:A:455:LEU:HD23	1.96	0.46
1:A:321:LYS:NZ	1:A:369:GLN:NE2	2.46	0.46
1:A:273:LEU:HD11	1:A:283:MET:HB2	1.97	0.45
1:B:283:MET:HE3	1:B:291:ARG:HD2	1.99	0.45
1:A:281:VAL:HG22	1:A:295:LYS:HA	1.98	0.45
1:A:386:ASP:CG	1:A:388:ARG:NH1	2.70	0.45
1:B:356:LYS:O	1:B:359:ARG:NH1	2.50	0.44
1:A:262:ILE:HG12	1:A:327:ALA:HB1	1.99	0.44
1:B:480:ARG:HD2	1:B:499:TRP:HB3	1.99	0.43
1:A:274:GLY:HA3	2:A:601:TAW:C11	2.48	0.42
1:A:264:ARG:HB3	1:A:329:VAL:HG11	2.02	0.42
1:A:491:LEU:O	1:A:495:MET:HG2	2.20	0.42
1:B:281:VAL:HA	1:B:295:LYS:HA	2.01	0.42
1:B:268:ARG:HH11	1:B:268:ARG:HG3	1.85	0.41
1:A:332:GLU:H	1:A:332:GLU:CD	2.24	0.41
1:A:263:PRO:O	1:A:266:SER:OG	2.34	0.41
1:B:323:VAL:CG2	1:B:339:GLU:HG2	2.43	0.40
1:B:446:TRP:CE3	1:B:499:TRP:HA	2.56	0.40

All (1) symmetry-related close contacts are listed below. The label for Atom-2 includes the symmetry operator and encoded unit-cell translations to be applied.

Atom-1	Atom-2	Interatomic distance (Å)	Clash overlap (Å)
1:B:397:ASN:OD1	1:B:438:ARG:NH1[1_655]	2.19	0.01

5.3 Torsion angles (i)

5.3.1 Protein backbone (i)

In the following table, the Percentiles column shows the percent Ramachandran outliers of the chain as a percentile score with respect to all X-ray entries followed by that with respect to entries of similar resolution.

The Analysed column shows the number of residues for which the backbone conformation was analysed, and the total number of residues.

Mol	Chain	Analysed	Favoured	Allowed	Outliers	Percentiles	
1	A	248/286 (87%)	244 (98%)	4 (2%)	0	100	100

Continued on next page...

CONFIDENTIAL VALIDATION REPORT

Continued from previous page...

Mol	Chain	Analysed	Favoured	Allowed	Outliers	Percentiles	
1	B	237/286 (83%)	231 (98%)	6 (2%)	0	100	100
All	All	485/572 (85%)	475 (98%)	10 (2%)	0	100	100

There are no Ramachandran outliers to report.

5.3.2 Protein sidechains (i)

In the following table, the Percentiles column shows the percent sidechain outliers of the chain as a percentile score with respect to all X-ray entries followed by that with respect to entries of similar resolution.

The Analysed column shows the number of residues for which the sidechain conformation was analysed, and the total number of residues.

Mol	Chain	Analysed	Rotameric	Outliers	Percentiles	
1	A	211/245 (86%)	211 (100%)	0	100	100
1	B	210/245 (86%)	208 (99%)	2 (1%)	76	90
All	All	421/490 (86%)	419 (100%)	2 (0%)	88	95

All (2) residues with a non-rotameric sidechain are listed below:

Mol	Chain	Res	Type
1	B	442	LYS
1	B	453	THR

Sometimes sidechains can be flipped to improve hydrogen bonding and reduce clashes. All (4) such sidechains are listed below:

Mol	Chain	Res	Type
1	B	324	GLN
1	B	513	GLN
1	A	369	GLN
1	A	513	GLN

5.3.3 RNA (i)

There are no RNA molecules in this entry.

CONFIDENTIAL VALIDATION REPORT

CONFIDENTIAL VALIDATION REPORT

5.4 Non-standard residues in protein, DNA, RNA chains (i)

There are no non-standard protein/DNA/RNA residues in this entry.

5.5 Carbohydrates (i)

There are no monosaccharides in this entry.

5.6 Ligand geometry (i)

2 ligands are modelled in this entry.

In the following table, the Counts columns list the number of bonds (or angles) for which Mogul statistics could be retrieved, the number of bonds (or angles) that are observed in the model and the number of bonds (or angles) that are defined in the Chemical Component Dictionary. The Link column lists molecule types, if any, to which the group is linked. The Z score for a bond length (or angle) is the number of standard deviations the observed value is removed from the expected value. A bond length (or angle) with $|Z| > 2$ is considered an outlier worth inspection. RMSZ is the root-mean-square of all Z scores of the bond lengths (or angles).

Mol	Type	Chain	Res	Link	Bond lengths			Bond angles		
					Counts	RMSZ	$\#\|Z\| > 2$	Counts	RMSZ	$\#\|Z\| > 2$
2	TAW	A	601	-	31,34,34	4.45	7 (22%)	33,48,48	2.45	7 (21%)
2	TAW	B	601	-	31,34,34	4.66	7 (22%)	33,48,48	2.68	9 (27%)

In the following table, the Chirals column lists the number of chiral outliers, the number of chiral centers analysed, the number of these observed in the model and the number defined in the Chemical Component Dictionary. Similar counts are reported in the Torsion and Rings columns. '-' means no outliers of that kind were identified.

Mol	Type	Chain	Res	Link	Chirals	Torsions	Rings
2	TAW	A	601	-	-	2/12/28/28	0/4/4/4
2	TAW	B	601	-	-	4/12/28/28	0/4/4/4

All (14) bond length outliers are listed below:

Mol	Chain	Res	Type	Atoms	Z	Observed(Å)	Ideal(Å)
2	B	601	TAW	N4-N3	-23.60	1.08	1.37
2	A	601	TAW	N4-N3	-21.82	1.10	1.37
2	A	601	TAW	C14-C13	-6.33	1.04	1.19
2	A	601	TAW	C1-N1	5.17	1.40	1.32
2	B	601	TAW	C1-N1	5.11	1.40	1.32
2	B	601	TAW	C12-C11	4.70	1.53	1.30

Continued on next page...

Continued from previous page...

Mol	Chain	Res	Type	Atoms	Z	Observed(Å)	Ideal(Å)
2	A	601	TAW	C12-C11	4.70	1.53	1.30
2	B	601	TAW	C14-C13	-4.03	1.10	1.19
2	B	601	TAW	C1-N2	3.57	1.40	1.33
2	A	601	TAW	C1-N2	3.52	1.40	1.33
2	A	601	TAW	C3-C2	-3.44	1.33	1.43
2	B	601	TAW	C3-C2	-3.39	1.34	1.43
2	B	601	TAW	C9-C6	-2.61	1.48	1.54
2	A	601	TAW	C5-C13	-2.30	1.40	1.43

All (16) bond angle outliers are listed below:

Mol	Chain	Res	Type	Atoms	Z	Observed(^{o})	Ideal(^{o})
2	B	601	TAW	C5-N4-N3	10.53	111.86	104.32
2	A	601	TAW	C5-N4-N3	9.66	111.23	104.32
2	B	601	TAW	N1-C1-N2	-6.41	118.67	128.68
2	A	601	TAW	N1-C1-N2	-6.27	118.88	128.68
2	B	601	TAW	C21-O2-C19	-3.99	108.84	117.51
2	B	601	TAW	C3-C4-N6	-3.42	116.49	122.67
2	A	601	TAW	C3-C4-N6	-3.33	116.65	122.67
2	B	601	TAW	C22-O3-C17	-3.29	110.36	117.51
2	B	601	TAW	C12-C11-C10	-3.25	114.69	121.33
2	A	601	TAW	C12-C11-C10	-3.22	114.75	121.33
2	A	601	TAW	C21-O2-C19	-2.84	111.35	117.51
2	A	601	TAW	C22-O3-C17	-2.79	111.45	117.51
2	B	601	TAW	C5-C3-C2	-2.31	102.21	106.55
2	B	601	TAW	C20-C15-C14	-2.17	116.21	120.19
2	A	601	TAW	C5-C3-C2	-2.13	102.55	106.55
2	B	601	TAW	C8-N5-C9	-2.04	106.27	111.55

There are no chirality outliers.

All (6) torsion outliers are listed below:

Mol	Chain	Res	Type	Atoms
2	B	601	TAW	C18-C19-O2-C21
2	B	601	TAW	C20-C19-O2-C21
2	B	601	TAW	C16-C17-O3-C22
2	B	601	TAW	C18-C17-O3-C22
2	A	601	TAW	C20-C19-O2-C21
2	A	601	TAW	C18-C19-O2-C21

There are no ring outliers.

CONFIDENTIAL VALIDATION REPORT

1 monomer is involved in 1 short contact:

Mol	Chain	Res	Type	Clashes	Symm-Clashes
2	A	601	TAW	1	0

The following is a two-dimensional graphical depiction of Mogul quality analysis of bond lengths, bond angles, torsion angles, and ring geometry for all instances of the Ligand of Interest. In addition, ligands with molecular weight > 250 and outliers as shown on the validation Tables will also be included. For torsion angles, if less then 5% of the Mogul distribution of torsion angles is within 10 degrees of the torsion angle in question, then that torsion angle is considered an outlier. Any bond that is central to one or more torsion angles identified as an outlier by Mogul will be highlighted in the graph. For rings, the root-mean-square deviation (RMSD) between the ring in question and similar rings identified by Mogul is calculated over all ring torsion angles. If the average RMSD is greater than 60 degrees and the minimal RMSD between the ring in question and any Mogul-identified rings is also greater than 60 degrees, then that ring is considered an outlier. The outliers are highlighted in purple. The color gray indicates Mogul did not find sufficient equivalents in the CSD to analyse the geometry.

Ligand TAW A 601

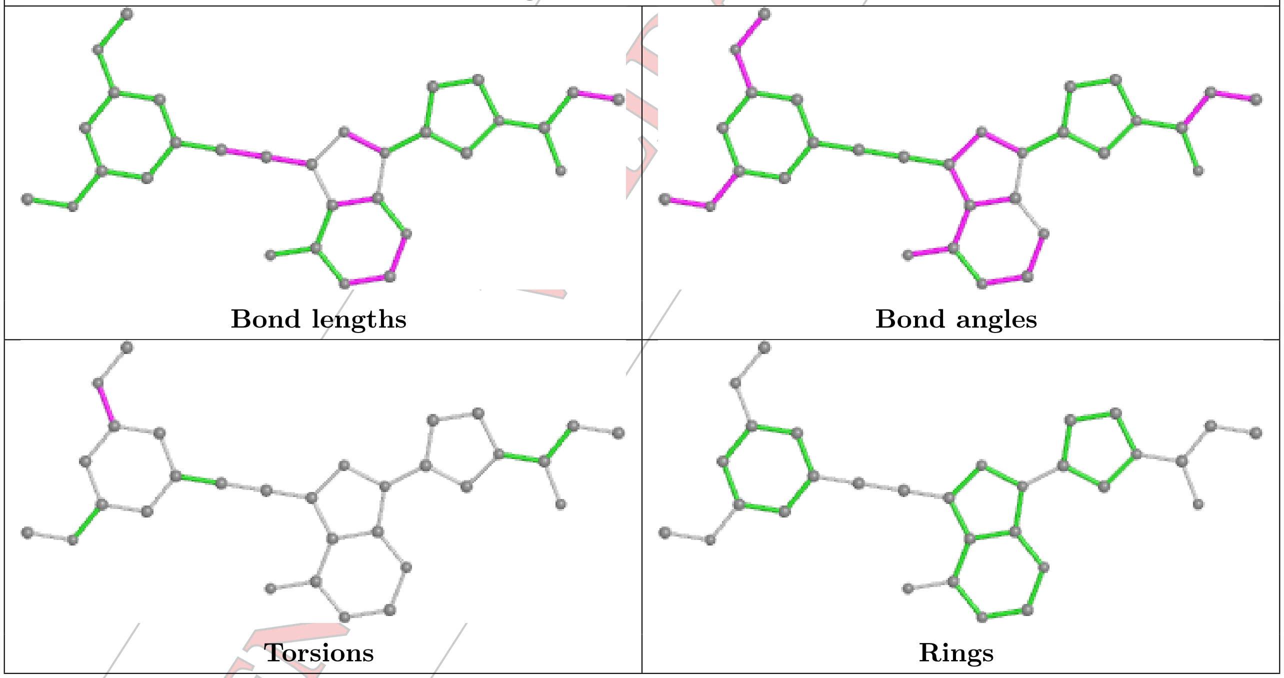

CONFIDENTIAL VALIDATION REPORT

Ligand TAW B 601

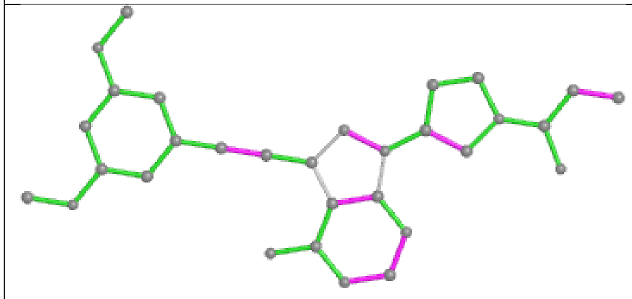

Bond lengths

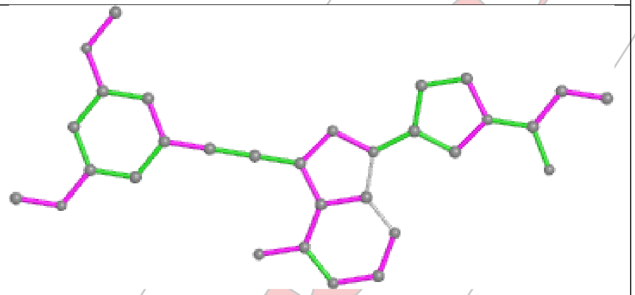

Bond angles

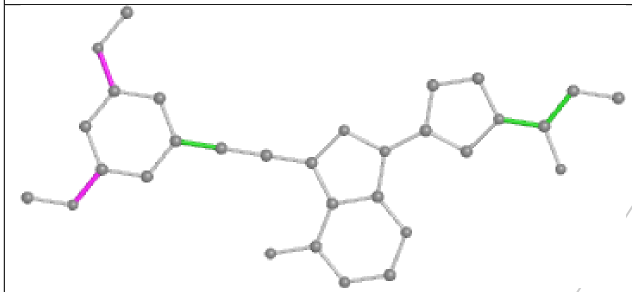

Torsions

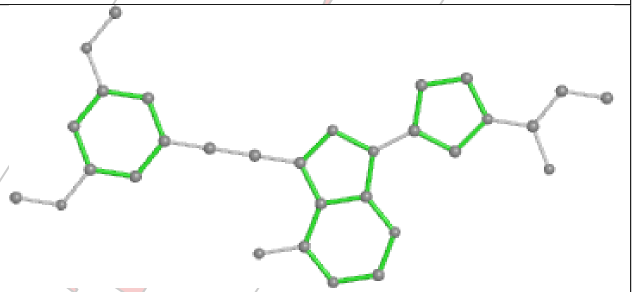

Rings

5.7 Other polymers (i)

There are no such residues in this entry.

5.8 Polymer linkage issues (i)

There are no chain breaks in this entry.

6 Fit of model and data (i)

6.1 Protein, DNA and RNA chains (i)

In the following table, the column labelled '#RSRZ> 2' contains the number (and percentage) of RSRZ outliers, followed by percent RSRZ outliers for the chain as percentile scores relative to all X-ray entries and entries of similar resolution. The OWAB column contains the minimum, median, 95th percentile and maximum values of the occupancy-weighted average B-factor per residue. The column labelled 'Q< 0.9' lists the number of (and percentage) of residues with an average occupancy less than 0.9.

Mol	Chain	Analysed	<RSRZ>	#RSRZ>2			OWAB(Å^2)	Q<0.9
1	A	254/286 (88%)	-0.10	7 (2%)	53	52	27, 49, 92, 113	0
1	B	247/286 (86%)	-0.19	7 (2%)	53	52	28, 46, 88, 127	0
All	All	501/572 (87%)	-0.15	14 (2%)	53	52	27, 48, 91, 127	0

All (14) RSRZ outliers are listed below:

Mol	Chain	Res	Type	RSRZ
1	B	301	THR	5.2
1	A	300	GLY	4.1
1	B	424	PHE	3.8
1	B	298	LYS	3.6
1	B	271	VAL	3.5
1	A	305	GLU	2.8
1	A	274	GLY	2.7
1	A	526	GLN	2.6
1	A	271	VAL	2.6
1	B	279	GLY	2.5
1	A	299	PRO	2.4
1	A	301	THR	2.4
1	B	526	GLN	2.3
1	B	288	GLY	2.3

6.2 Non-standard residues in protein, DNA, RNA chains (i)

There are no non-standard protein/DNA/RNA residues in this entry.

6.3 Carbohydrates (i)

There are no monosaccharides in this entry.

CONFIDENTIAL VALIDATION REPORT

6.4 Ligands (i)

In the following table, the Atoms column lists the number of modelled atoms in the group and the number defined in the chemical component dictionary. The B-factors column lists the minimum, median, 95th percentile and maximum values of B factors of atoms in the group. The column labelled 'Q< 0.9' lists the number of atoms with occupancy less than 0.9.

Mol	Type	Chain	Res	Atoms	RSCC	RSR	B-factors($Å^2$)	Q<0.9
2	TAW	A	601	31/31	0.86	0.23	55,70,89,93	0
2	TAW	B	601	31/31	0.90	0.21	44,73,91,105	0

The following is a graphical depiction of the model fit to experimental electron density of all instances of the Ligand of Interest. In addition, ligands with molecular weight > 250 and outliers as shown on the geometry validation Tables will also be included. Each fit is shown from different orientation to approximate a three-dimensional view.

Electron density around TAW A 601:

2mF$_o$-DF$_c$ (at 0.7 rmsd) in gray
mF$_o$-DF$_c$ (at 3 rmsd) in purple (negative)
and green (positive)

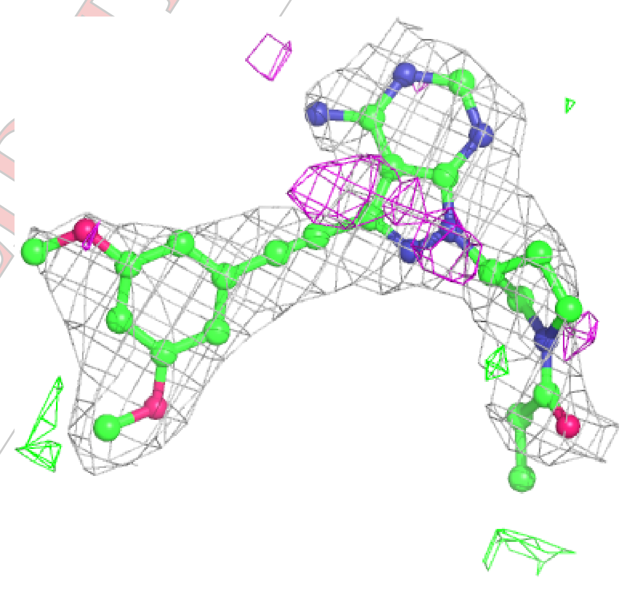

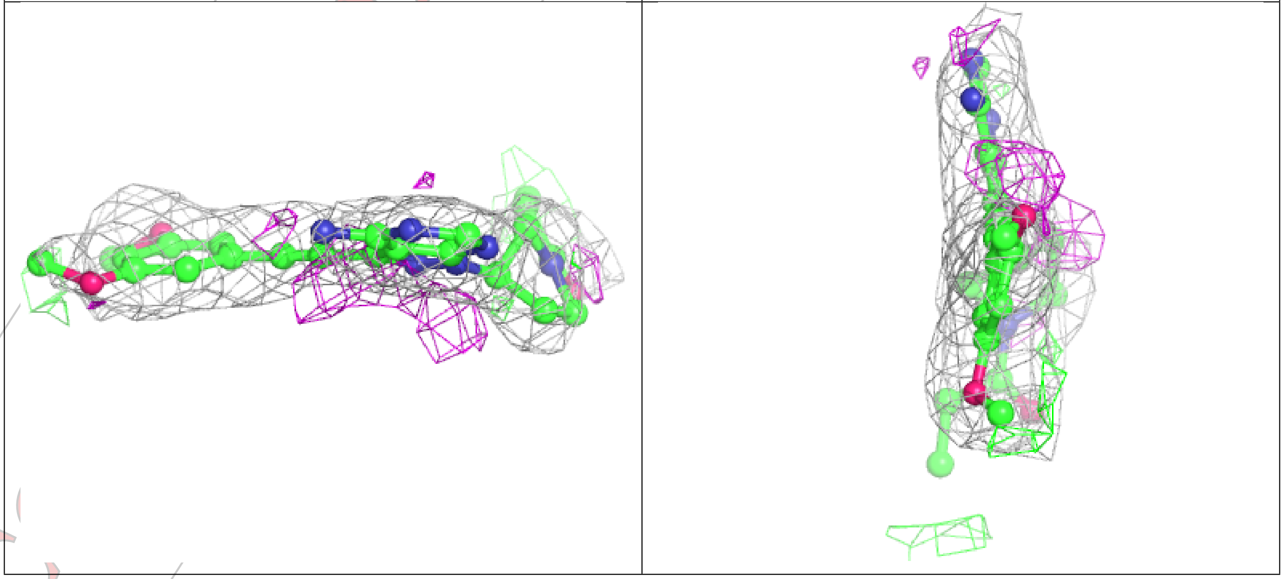

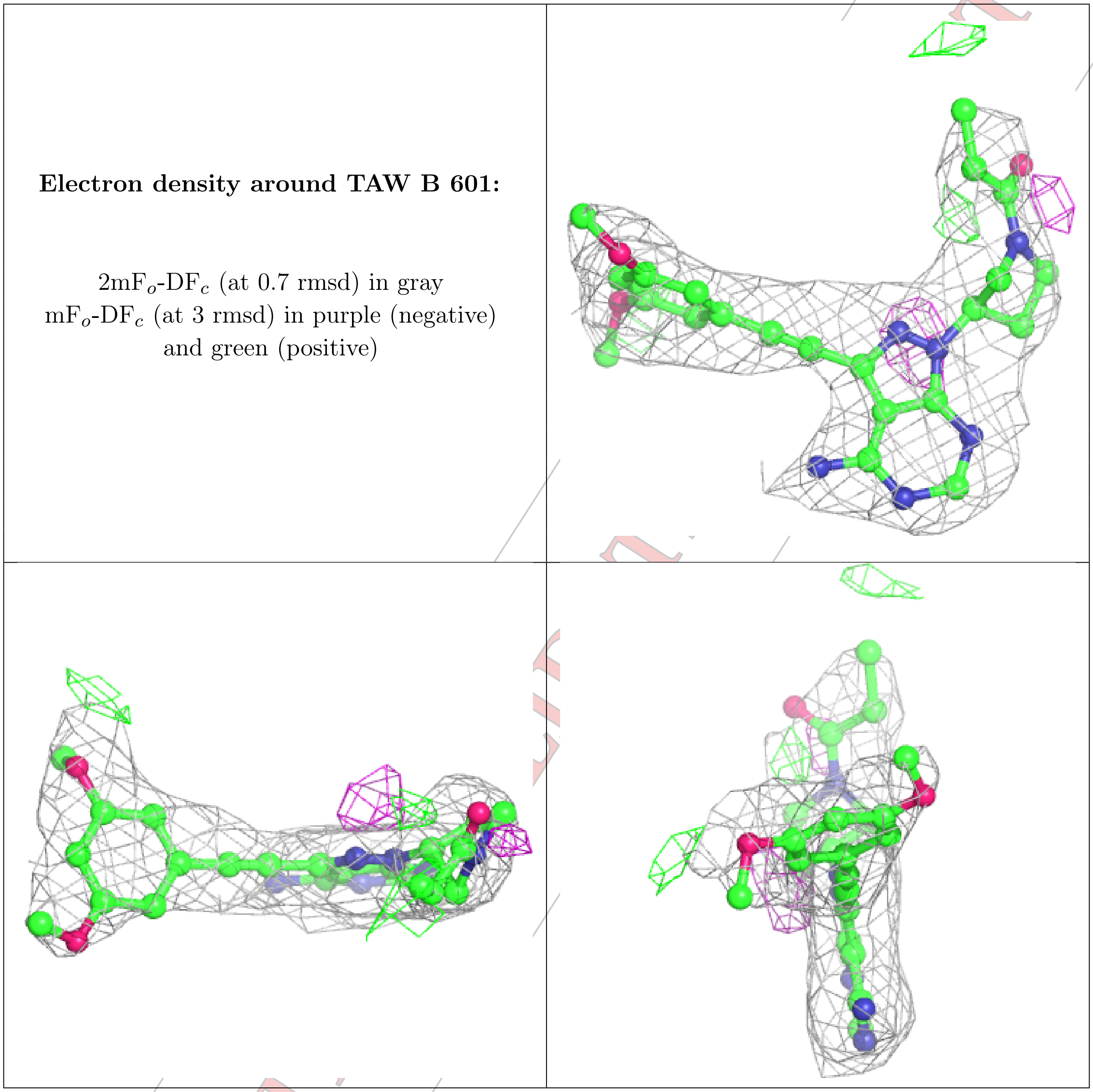

Electron density around TAW B 601:

$2mF_o$-DF_c (at 0.7 rmsd) in gray
mF_o-DF_c (at 3 rmsd) in purple (negative)
and green (positive)

6.5 Other polymers ⓘ

There are no such residues in this entry.

CONFIDENTIAL
